# A role of NLRP3 and MMP9 in migraine progression: a systematic review of translational study

**DOI:** 10.3389/fneur.2024.1307319

**Published:** 2024-05-21

**Authors:** Rapuru Rushendran, Anuragh Singh, S. Ankul Singh, Vellapandian Chitra, Kaliappan Ilango

**Affiliations:** ^1^Department of Pharmacology, SRM College of Pharmacy, SRM Institute of Science and Technology, Kattankulathur, Chengalpattu, Chennai, India; ^2^Department of Pharmaceutical Chemistry, Tagore College of Pharmacy, Chennai, India

**Keywords:** NLRP3, MMP9, migraine, IL1β, P2X7R, P2X4, IL1-β, IL-18

## Abstract

**Background:**

Migraines affect one billion individuals globally, with a higher occurrence among young adults and women. A significant survey in the United States indicated that 17.1% of women and 5.6% of men suffer from migraines. This study seeks to investigate the potential connection between NLRP3 and MMP9 in migraine pathology.

**Methods:**

The research involved searching databases such as PubMed, Scopus, Science Direct, Google Scholar, and Proquest, with the search concluding on March 31, 2024. Following PRISMA guidelines, PICO data were collected, focusing exclusively on animal models induced by Nitroglycerine (10 mg/kg), while excluding clinical studies.

**Results:**

The study, originally registered in Prospero Reg. No. CRD42022355893, conducted bias analysis using SYRCLE’s RoB tool and evaluated author consensus using GraphPad v9.5.1. Out of 7,359 search results, 22 papers met the inclusion criteria. Inter-rater reliability among reviewers was assessed using Cohen’s kappa statistics.

**Conclusion:**

This review summarizes 22 preclinical studies on Nitroglycerin (NTG), NLRP3, MMP9, and related biomarkers in migraine. They reveal that NTG, especially at 10 mg/kg, consistently induces migraine-like symptoms in rodents by activating NLRP3 inflammasome and stimulating proinflammatory molecule production.

**Systematic Review Registration:**

https://www.crd.york.ac.uk/prospero/, CRD42022355893.

## Graphical abstract

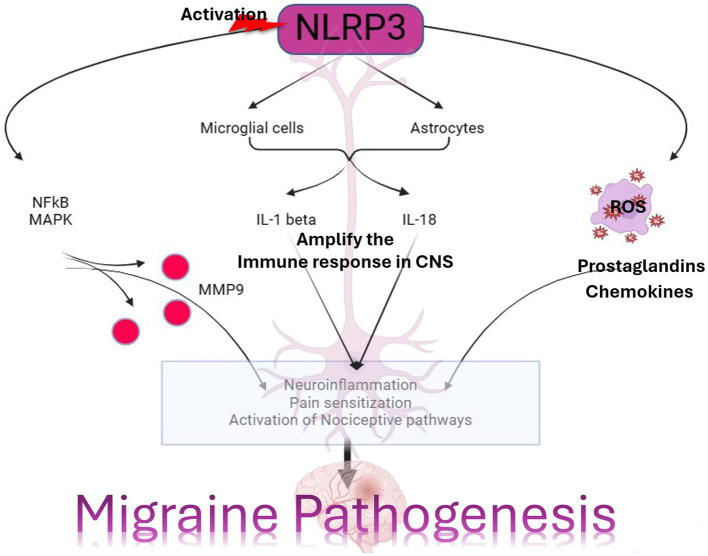

## 1 Introduction

One of the most common causes for outpatient visits to general neurologists is migraine, a prevalent and incapacitating neurological condition. Lifestyle changes are a cornerstone of treatment in addition to pharmaceutical therapy (1). Scientists have yet to find a medication that treats migraine for ten decades. They started targeting different biomarkers such as NFkB (2–4), interleukins (5–7), serotonin (8–11), etc. which was released immensely in the migraineurs but unable to find a way to cure. Then the treatment for migraine has changed dramatically as a result of medications that target the CGRP pathway (12–14). Numerous articles have provided evidence for the efficacy of monoclonal antibodies targeting CGRP or its receptor in preventing difficult-to-treat migraines (15–19). A trial of intravenous eptinezumab in individuals with 2–4 prior failed efforts at preventive therapy was conducted in 2022 to supplement this work (20–22). During the 12-week double-blind observation period, a single infusion of 300 mg eptinezumab significantly reduced the number of monthly migraine attack days compared to the placebo group (23–25). Nowadays, researchers are especially targeting NLRP3 inflammasomes (26–30) which has a robust connection to migraine and predicting MMP9 additionally playing a major role in the migraine attack (31–33).

Neurological diseases, whether fatal or non-fatal, constitute a significant portion of the non-communicable disease burden in India (34). According to the 2019 Global Burden of Disease study (GBD2019), migraine was reported as the second leading cause of disability, especially among women under the age of 50. Out of 357 publications primarily from high-income countries, the estimated global prevalence of active headache disorders was 52.0% (with a 95% confidence interval of 48.9–55.4). Specifically, the prevalence of migraine was estimated at 14.0% (with a confidence interval of 12.9–15.2), tension-type headache (TTH) at 26.0% (with a confidence interval of 22.7–29.5), and other severe headache disorders (H15+) at 4.6% (with a confidence interval of 3.9–5.5) (35). These estimates aligned with the figures for migraine and tension-type headache (TTH) reported in GBD2019, the most recent available data, but they indicated a higher prevalence of headaches in the general population. Specifically, on a daily basis, 15.8% of the global population experiences headaches (35, 36). A statistical report from 2019 found that headache disorders, including conditions like migraine and tension-type headaches, were the most common neurological conditions, affecting approximately 488 million individuals in India (with a 95% uncertainty interval ranging from 449 to 527 million) (37). Regardless of regional differences, the issue of headaches is a global concern, affecting people of various ages, ethnicities, income levels, and geographic locations. Between half to three-quarters of individuals aged 18 to 65 worldwide experienced a headache in the past year, with 30% or more reporting migraine of the two main categories of headache disorders, migraine has the most significant impact on overall neurological health, contributing the highest share of Disability-adjusted life years (DALYs) (38). The DALYs associated with migraine significantly surpass those linked to tension-type headaches. Between 1990 and 2019, India saw an increase in the raw prevalence and DALY rate of headache disorders, while the age-standardized prevalence and DALY rate remained relatively stable. In 2019, it was observed that females between the ages of 35 and 59 had a higher prevalence of migraine compared to males in the same age group (39).

The prevalence of this condition increases with age, peaking between 40 and 44 years, and then gradually decreases in both men and women. The proportion of non-communicable neurological disorders was 4.0% (with a 95% uncertainty interval of 3.2–5.0) in 1990, doubling to 8.2% (with an interval of 6.6–10.2) by 2019. Projections indicate that this percentage is expected to climb to 16.5% by 2040. Migraine, a widespread neurological disorder, stands as the second most significant contributor to years lived with disability globally and ranks among the top twenty most disabling conditions worldwide (36, 40–42). Migraine is a severe neurological condition diagnosed through clinical criteria (43). Many individuals are unable to work due to migraines, which are complex neurological events lasting for several hours or even extending over multiple days. The most common type of migraines is those without an aura (44). As a result, it is estimated to affect hundreds of millions annually (45). The underlying processes in migraine pathophysiology have a significant genetic component and involve the activation of pain pathways associated with the trigeminovascular system (46–51). This systematic study is motivated by the exploration of the roles of NLRP3 and MMP9 in the onset of migraine attacks. After identifying, evaluating, and consolidating research results, a thorough examination of relevant preclinical studies is essential to establish the reliability and potential applicability of the data in future research. This hypothesis sets the stage for establishing a robust connection between NLRP3 and MMP9 in the context of migraines.

## 2 Methods

Three authors independently evaluated the inclusion and exclusion criteria to reduce the chance of excluding pertinent records and determine whether the studies were eligible. After removing duplicate entries, the title, and abstract of the remaining records were evaluated in the first screening. The analysis included NLRP3 and MMP9 biomarker studies assessing the relationship with the progression of chronic migraine pathology through pain pathways. No limitations on the length of the survey, its follow-up, or the date of publication have been imposed. The study excluded *in vitro* and *in vivo* animal experiments, narrative or systematic reviews, meta-analyses, abstracts, proceedings, conference communications, editorials, and book chapters. Additionally, publications that were either unavailable in full text/not published in English were excluded, as indicated in Table 1. This systematic review adhered to the guidelines outlined in the Preferred Reporting Items for Systematic Review and Meta Analysis Protocols (PRISMA-P). The review followed the Population, Intervention, Comparison, and Outcome strategy (52). The following were the PICO requirements for inclusion.

**Table 1:** Inclusion and Exclusion criteria.

Aspects of research	Inclusion	Exclusion
Population	Rodents	Non-Rodents
Interventions	New strategies with the express purpose of alleviating migraine symptoms in preclinical studies. NLRP3 and MMP9 biomarkers and other proinflammatory molecules are involved in it.	Migraine trails in clinical point of view.
Induction model	Nitroglycerine inducing model	Other than the nitroglycerine induction animal model; Comorbities related to the Nitroglycerine induction model such as Cardiovascular, Endocrine, and Psychiatric disorders.
Language	English	Non-English
Outcome	Any	Other than rats and mice
Comparators	Any	Other than rats and mice

*Population*: All species of rats and mice; there are no restrictions for selection of age and gender.

*Intervention*: Nitroglycerin-inducing animal models were explicitly focused on in this study. The role of biomarkers such as NLRP3 and MMP9 are scrutinized in all the studies of migraine.

*Comparison*: The non-clinical model of Nitroglycerine induction will be included, but groups with comorbidities will be excluded from the analysis.

*Outcome:* Behavioral test, Sensory sensitivity testing, von Frey Testing, Thermal Withdrawal Latency Test, Paw Licking Time Test, Gelatin gel zymography, Light/dark test, Von Frey test, Hot plate test, Orofacial formalin test, immunohistochemistry, Histopathology, Immunofluorescence staining, Flow Cytometry, qRT PCR will be included in this review. Different Outcomes will be excluded from scrutiny if they have no bearing on the development of migraine. Inclusion was not restricted to a specific period, and only original research published in English and reviewed by academic peers was considered.

### 2.1 Search strategy

We conducted a literature search by consulting the most pertinent scientific databases, including PubMed, Science Direct, Scopus, Google Scholar, and Proquest. There were no limitations on the publication date. The search encompassed records matching the specified search terms from the inception of these databases up to March 31, 2024, which was the date of the last search. To initiate the review, we primarily used Pub Med,^1^ a search engine managed by the National Center for Biotechnology Information of the US National Library of Medicine, under the National Institutes of Health. Filters on PubMed were utilized to narrow the results down to human clinical trials and literature available in English. The results were not restricted by publication date, and the last search was performed on March 31, 2024. We employed the search terms (NLRP3 AND Migraine), (MMP9 AND Migraine), (Preclinical AND Migraine), (Migraine), and (Herbal treatment AND Migraine) across the PubMed, Science Direct, Google Scholar, Proquest, and Scopus search platforms.

### 2.2 Data retrieval

In this process, one reviewer (RR) collected data from each study, which encompassed information such as the animal strain, weight, route of administration (ROA), dose, and the specific biomarkers addressed in the study. This data was then documented in an Excel Sheet for the purpose of data management. Subsequently, three reviewers (ASS, AS and RR) cross-validated the gathered information to ensure its coherence and relevance.

### 2.3 Risk of bias

Using the Systematic Review Centre for Laboratory Animal Experimentation risk of bias (SYRCLE’s RoB) method (53–55), five reviewers (IK, CV, RR, ASS, and AS) conducted individual assessments of bias risk for all 22 studies. This assessment questionnaire comprises ten categories associated with six types of bias, evaluating the methodological quality of preclinical studies. These categories encompass elements like sequence generation, baseline characteristics, allocation concealment, random housing, random outcome assessment, incomplete outcome data, selective outcome reporting, and other factors, such as drug pooling, funder influence, design-specific bias risk, unit of analysis errors, and replacement of dropouts from the original population. Each study’s risk of bias was evaluated by all reviewers using the designations ‘yes’, ‘no,’ and ‘unclear’ to denote high risk, low risk, and insufficient information to determine the risk of bias, respectively. Any disparities in these assessments were resolved through discussion among the reviewers or by reaching a consensus. The author agreement is assessed based on kappa values with reference values of less than zero No agreement; 0.00–0.20 Slight agreement; 0.21–0.40 Fair agreement; 0.41–0.60 Moderate agreement; 0.61–0.80 Substantial agreement; 0.81–1.000 Almost perfect agreement. The level of agreement was quantified using kappa, employing the GraphPad by Dotmatics tool.^2^

### 2.4 Data analysis

This review was officially registered with PROSPERO under the reference CRD42022355893. We conducted a thorough examination of the literature to ascertain the extent and prevalence of the utilization of particular concepts, objectives, and correlated outcome measures in preclinical trials focused on migraine treatments. The methodology protocol adhered to the Preferred Reporting Items for Systematic Reviews and Meta-Analyses (PRISMA) guidelines (56), which offer consensus-based standards for developing and carrying out high-quality systematic literature reviews. The PRISMA checklist provides guidelines for various stages of conducting a literature search and review. This includes specifying eligibility criteria for published papers, determining the dataset for the search, formulating search terms, establishing a standardized review process for identified publications, implementing record tracking and data management systems, outlining the data to be extracted from each publication meeting inclusion criteria, and devising a strategy for data extraction from qualifying publications.

## 3 Results

### 3.1 Study selection

**Figure 1:**
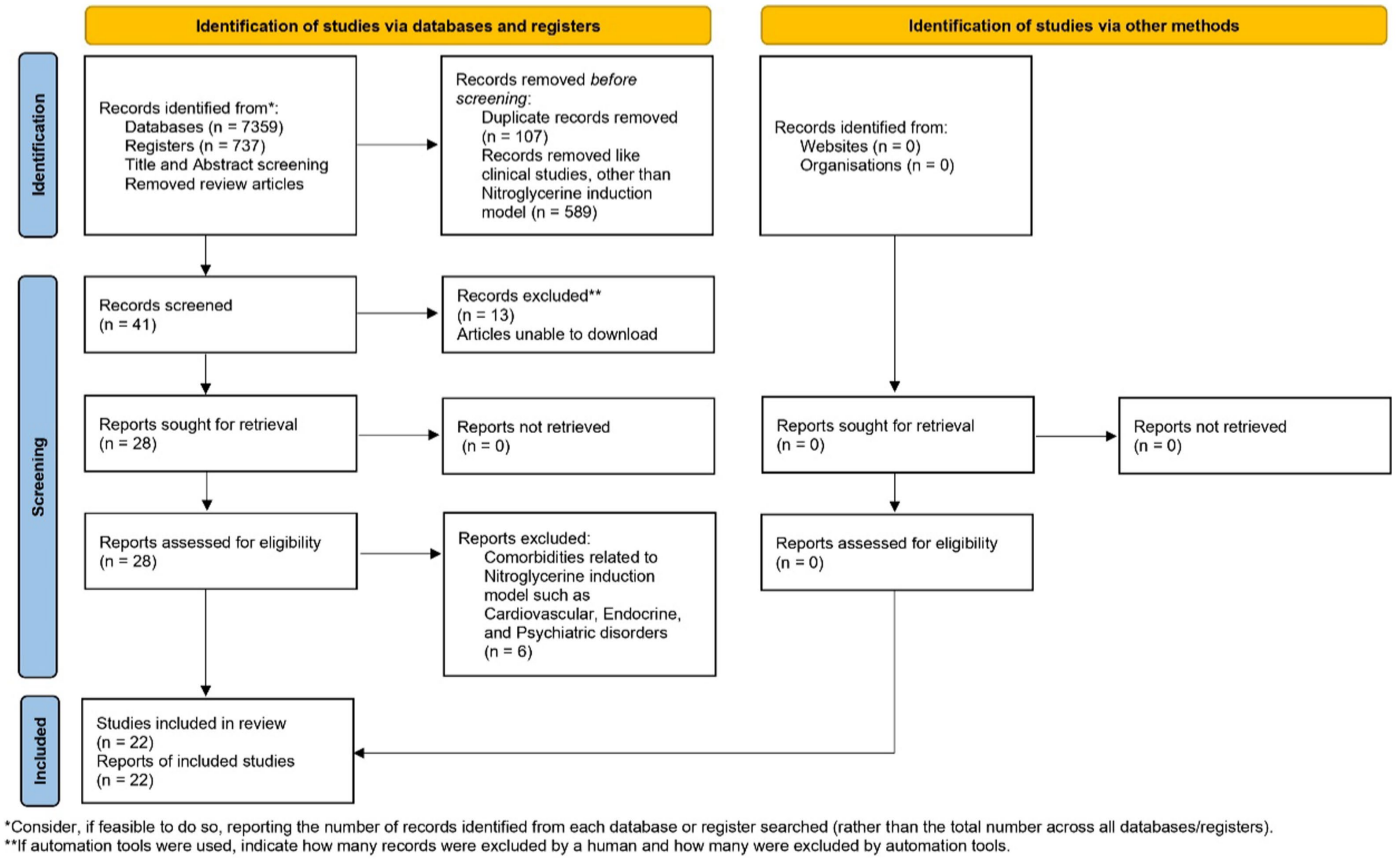
Schematic representation of article screening.

The database search yielded 7,359 results, with 47 records from PubMed, 323 from Science Direct, 803 from Scopus, 6,180 from Google Scholar, and 6 from Proquest. Initially, 737 titles were screened, and after eliminating duplicates, book chapters, other than NTG induction model, clinical studies, and conducting abstract screening, at last 22 remained. All of the studies that were included adhered to ethical guidelines and received approval from the relevant institutional bodies. The characteristics and outcomes of these selected studies are listed in Table 2. Various animal species were represented among the rodents in the study, with the Nitroglycerin (10 mg/kg, b.wt) model being the most frequently employed for inducing migraine-related conditions, and it was the primary focus. The timeline of the systematic review is presented in Figure 1.

**Table 2:** Characteristics of the included studies and a summary of the outcome.

Author/Year	Cell line/Animal Strain and weight	Induction	ROA/Dose	Biomarkers/Receptors/Cell culture	Endpoint	Outcome
Sun et al. (57)	C57BL/6 J Male 20-30 g BV2 cells	NTG induced	i.p 10 mg/kg	cfos, CGRP, p-65, NLRP3, Caspase-1, IL-1β, IL-18, GFAP, TREM-1, NeuN, Iba	Behavioral assessment Western blot Immunofluorescence	It shows that TREM1 regulates microglial and NLRP3 inflammasome activity via modulating the NF-κB signaling pathway. Furthermore, TREM1 was linked to CGRP and c-fos expression, contributing to central sensitization. These discoveries offer new insights into CM mechanisms, suggesting that targeting TREM1 may be a feasible treatment option.
Liu et al. (58)	C57BL/6 J Male 20-30 g	NTG induced	i.p 10 mg/kg	CGRP, cFOs, SOD, MDA, ROS intensity, iNOS, IL-6, IL-1β, TNF-α, Nrf2, HO-1, NQO1,	Light/dark test, Von frey test Elevated plus maze	Dl-3-n-butylphthalide (NBP) significantly reduced the severity of migraines in mice that were caused by NOG. Therapeutic results were achieved by lowering oxidative stress and neuroinflammation via activating the Nrf2 signaling pathway, which in turn reduced nociceptive hypersensitivity and central sensitization. As a result, NBP shows promise as a migraine prevention treatment.
Xu et al. (59)	Mice	NTG induced	i.p 10 mg/kg	ROS, MDA, SOD, NrF2, HO1, NQO1, MZF1	Behavior tests and immunofluorescence assay	Wuzhuyu Decoction down-regulated PGK1 via MZF1, activating the NRF2 pathway.
Lu et al. (60)	C57BL/6 J Mice Male 20-30 g	NTG induced	i.p 10 mg/kg	ATP, ADP AMP, IL-1β, IL-4, IL-6, IL-10, TNF-α, IFN-γ, CGRP, AMPK, PAMPK, UHRF1, iNOS, Arg1, Iba1, NFkB, P65, and GM-CSF	ELISA The Luminex liquid suspension chip assay Immunohistochemistry	Their findings strongly suggests that AMPK plays a crucial role in central sensitization of chronic migraine (CM), especially in cases of aberrant energy metabolism. AMPK activation reduces neuroinflammation in NTG-induced CM mice via polarizing microglia in M2. AMPK activity modulation may aid in treating CM by targeting central sensitization processes.
Xie et al. (61)	C57BL/6 J Mice Male 21-32 g	NTG induced	i.p 10 mg/kg	AT, MRC complex-I, Fundc1, Fis1, Mid49, OPA1, PGC1α, TFAM, CHOP, HSP10, MDA	Behavioral test, von-Frey filaments, a hot plate, and a light–dark box, Light-aversive test, qPCR, ELISA, TEM,	In the thalamus, hypothalamus, periaqueductal grey, trigeminal ganglio, and trigeminocervical complex, respectively, 529, 109, 163, 152, and 419 differentially expressed proteins were found using proteomics profiling of the CM model. Significant alterations in region-specific brain circuits indicated mitochondrial dysfunction in the thalamus. UA intervention had a modest attenuating effect on NTG-induced mitochondrial structural damage, malfunction, and homeostatic dysregulation.
Zhang et al. (62)	SPF-grade Wistar rats 250-300 g	NTG induced	i.p 10 mg/kg	Fibrinogen, Tail lift index, Negative geotaxis, Air righting reflex, vestibular dysfunction ratings	Behavioral tests ELISA	Their findings demonstrated that rats subjected to NTG-induced chronic migraines exhibited decreased vestibular function. After several injections of NTG, the fibrinogen levels rose. The rats used to study migraines showed no changes in mechanical hyperalgesia (either worsening or improving) or vestibular dysfunction (either improving or worsening) after defibrinogenation.
Zhang et al. (62)	Male SD Rats 210-235 g	NTG induced	i.p 10 mg/kg	pCREB, PACAP, PAC1R, ERK, BDNF, cFos	Behavioral test Immunofluroscence staining Western blot TEM	Overall, the research work found to be the expression of PACAP and PAC1R in TNC was elevated after repeated treatment of NTG to rats. In addition, via modifying NTG-induced synaptic plasticity via the ERK/CREB/BDNF pathway, PACAP6-38, a selective PAC1R antagonist, reduced chronic cephalic allodynia and inhibited the augmentation of neuronal activity. Inhibiting PACAP/PAC1R could be a new therapeutic target for migraine based on our findings.
Qi et al. (63)	Wistar rats Male 200-250 g	NTG induced	i.p 10 mg/kg	–	ABR Latency	The postauricular nitroglycerin injection is safer and more effective than intraperitoneal injection for migraine modeling in rats. Postauricular nitroglycerin injection damages rats hearing function more. The migraine model rat caused by nitroglycerin postauricular injection may represent a new cochlear migraine model.
Kim et al. (64)	C57BL/6 Male	NTG induced	i.p 10 mg/kg	cFos expression; cold allodynia measurements in the hindpaws and facial region	Immunohistochemistry	When repeatedly stimulated at the GV16 acupoint, mice with NTG-induced peripheral hypersensitivity and high TNC c-Fos expression can reduce hindlimb and face mechanical and cold allodynia. DBV injections at GV16 activate alpha-2 adrenoceptors, not endogenous opioid receptors. Based on this study, chemotherapeutic acupuncture with Diluted Bee Venom at GV16 may help migraine patients with peripheral discomfort.
Xu et al. (65)	C57BL/6 mice Male 7 weeks old	NTG induced PC12 cell line	i.p 10 mg/kg	ROS, c-Fos, NeuN, PTEN, NQO1, PGK1, SOD, MDA, and NRF2	Behavioral tests Cell viability assay Western blot	Their study demonstrated that RUT reduced oxidative stress and relieved migraine symptoms by blocking PGK1 activity with PTEN and activating the Nrf2 antioxidant mechanism. Based on these results, RUT, a natural medicine, may be a good choice for migraine treatment.
Luo et al. (66)	SD rats	NTG induced	i.p 10 mg/kg	COX-2, PGE2, CGRP, ERK, SRC, TRPV1	Elisa, rtPCR, Western blot	Shaoyao Gancao Decoction considerably inhibits the NGF/TRPV1/COX-2 signaling pathway, which is responsible for central hyperalgesia migraine. This indicates that the molecular mechanism by which SGD alleviates migraine symptoms might be associated with the central hyperalgesia neurotransmitter, which controls the pathophysiology of migraine.
Zhang et al. (62)	SD rats Male 200–250 g	NTG induced	i.p 10 mg/kg	c-Fos, CGRP, PACAP, PAC1, PKA and ERK	Von Frey filaments, and hot plate tests, Immunofluorescence, Western blot	Their investigation showed that repeated intranasal PACAP treatments caused mechanical and thermal hyperalgesia like NTG injections. The impact of PACAP on migraine attacks involves PAC1 receptor internalization and PKA and ERK signaling pathways. This is the first research to show that inhibiting PACAP-induced PAC1 receptor internalization improves hyperalgesia in CM rats by restricting ERK signaling. PACAP-induced trigeminal vascular activation requires further study to determine the process and uncover additional targets that modulate PAC1 receptor internalization with specificity.
Zhai et al. (67)	SD rats 230–250 g Male	NTG induced	50 μg/kg and 100 μg/kg perampanel	PKC PLC PKA CREB PACAP	ELISA Western blot	Perampanel inhibited PACAP expression in an *in vitro* research by inhibiting the cAMP/PKA/CREB pathway. However, the mechanism by which perampanel affects the cAMP/PKA/CREB pathway is complex and uncertain. Studying the protective benefits of perampanel against diseases like migraine can shed light on the causes of nervous system problems and aid in developing effective treatments.
Mason et al. (68)	ICR mice PAR2KO mice Male and female 5–8 weeks	NTG induced	10 μL /g	IL6	Mechanical hypersensitivity and grimace assays Facial hypersensitivity Mouse grimace scale	They demonstrate that PAR2 activation and IL-6 signaling can prime a NO donor through different methods. Targeting IL-6 in people is currently being tested, but its efficacy in migraine has yet to be determined. This study, together with previous research, strongly suggests that PAR2 may be a promising therapeutic target for migraine.
Ge et al. (69)	SD rats Male 200 ± 10 g	NTG induced	i.p 10 mg/kg	CGRP cfos	Immunohistochemistry Behavioral tests	The integrated approach combining harmacodynamics, metabolomics, and network pharmacology to understand the impact and mechanism of MXFD on migraine. Our integrated analysis revealed two differentially expressed metabolites, 5-MIAA and DCA, and nine targets, including MAOB, MAOA, ADRB1, ADRB2, ADRB3, ADORA2A, ADORA2B, DRD5, and HTR4. MXFD’s effectiveness in treating migraine is significantly impacted by these metabolites and targets. This research provides useful data for exploring the mechanisms behind MXFD’s therapeutic effects.
A. Filippone et al. (27)	CD1 female and male mice 25-30 g	NTG induced migraine model	i.p 10 mg/kg	NLRP3 IL-18 IL-1β TNF-α NGF BDNF NT-3	Light/dark test, Von frey test, Hot plate test, Orofacial formalin test, ELISA, Immunofluorescence, RT-qPCR, and Histological analysis	Neuronal damage; IL-18, IL-1β, and TNF-α protein expressions; mRNA expressions of these proteins; Nerve growth factor (NGF), Brain-derived neurotropic factor (BDNF), and Neurotrophin-3 (NT3) expressions were dramatically decreased when treated with BAY-117082 at 5 mg/kg and 10 mg/kg. These report suggesting that it significantly modulated Erk/CREB/Akt pathways and could be considered novel strategy therapeutics for migraine treatment.
Pan et al. (70)	C57BL6/J mice 20-25 g	NTG induced	i.p 10 mg/kg	S1PR1 STAT3	Behavioral test qRT-PCR Western blot Immunofluorescence staining	W146 (3-amino-4-(3-hexylphenylamino)-4-oxobutylphosphonic acid) could alleviate chronic NTG induced hypersensitivity and reduce the expression of CGRP, pSTAT3 and c-fos in the Trigeminocervical complex by blocking S1PR1 activity.
Aral et al. (31)	Neonatal mouse	GTN induced	–	CRLR/CGRPR1 MMP9	qPCR, Perl’s histochemistry, Immunofluorescence staining and ELISA.	Elevation of nitric oxide which has been demonstrated to have a role in the production of inflammation such as MMP9 and CGRPR1 may regulate cellular iron traffic; hence, different cells and mislocalization of iron within the cell may contribute to migraine pathophysiology via diverse molecular mechanisms.
Ting et al. (71)	C57BL/6 mice 20-25 g	NTG induced migraine model	i.p 10 mg/kg	IL-1β IL-18 CGRP NLRP3	Behavioral tests Rota-rod test Western blot qRT-PCR Immunofuorescence staining	BoNT/A suppressed the expression of the NLRP3 protein and the pro-inflammatory cytokine IL-1β in the TNC produced by NTG, and it may regulate the activity of microglia in the TNC through modulating CGRP. It is possible to interact with microglia, which controls the release of inflammatory factors but, BoNT/A therapy had no effect on LPS-induced pro-inflammatory factor release in astroglia.
Wang et al. (72)	Mice 20 ± 2 g Rat 200-266 g	NTG induced migraine model	i.p 10 mg/kg	CGRP Substance P CCK 5-HT COX-2 CB1R	ELISA Immunohistochemical analysis	. Hejie Zhitong prescription decreases the production of neurotransmitters linked with nociceptive transmission, including as 5-HT, CGRP, and CCK, which may be connected to its overexpression of CB1R and downregulation of COX-2.
He et al. (28)	Male C57BL/6 mice 8–12 weeks old 18-25 g	NTG induced migraine model	i.p 10 mg/kg	c-Fos CGRP p-ERK NLRP3 IL-1β	Sensory sensitivity testing, Western blot, qRT-PCR and Immunofuorescence staining	Both NLRP3 and IL-1 inhibition alleviated hyperalgesia and decreased the increase in biomarkers such as p-ERK, c-Fos and CGRP associated with central sensitization of CM in the TNC. The NLRP3 inflammasome may be a target for controlling CM-associated pain, and inhibiting it may represent a novel therapeutic rationale and technique for migraine treatment.
Kim et al. (73)	Male Sprague Dawley rats 270-300 g	GTN	i.v 10 μg/kg	COX-2 TNF-α MMP9	Western blot analysis and Immunohistochemistry	Proinflammatory mediators such as COX-2, TNF-α, and MMP9 play a role in the NO-mediated migraine pathogenesis cascade. Further research into these inflammatory mediators in the various transcription factor pathways may have pharmacological implications for new migraine therapies.

NLRP, Nod like receptor; IL-Interleukin; MMP, Matrix metalloproteinase; TNF, Tumor necrotic factor; BDNF, Brain derived neurotropic factor; NO, Nitric oxide; RAGE, Receptor for advanced glycation end product; COX, Cyclooxygenase; HMGB, High mobility group box; MAPK, Mitogen activated protein kinase; NFkB, Nuclear factor kappa B; TIMP, Tissue inhibitor of metalloproteinase; ERK, Extracellular signal-regulated kinase; CGRP, Calcitonin gene related peptide; CCK, Cholecystokinin; 5-HT, Serotonin; Iba, Ionized calcium binding adaptor molecule; ROS, Reactive oxygen species; TXNIP, Thioredoxin-interacting protein; TRPA, Transient receptor potential ankyrin; P2X4, P2X purinoceptor 4; P2X7R, P2X purinoceptor 7 receptor; S1PR1, Sphingosine-1-phosphate receptor 1; STAT, Signal transducer and activator of transcription; CXCL1, The chemokine (C-X-C motif) ligand 1; CCL2, chemokine (C-C motif) ligand 2; NGF, Nerve growth factor; NT3, Neurotrophin-3; i.p, Intraperitoneal; s.c, Subcutaneous; i.v, intravenous; p.o, Per oral; NTG, Nitroglycerin; GTN, Glyceryl trinitrate; CCI, Chronic constriction injury; LPS, Lipopolysaccharide; CSD, Cortical spreading depression.

### 3.2 Population

Albino Sprague Dawley rats and mice, CD1 mice, Wistar rats, Knockout mutant mice, Neonatal Mice, and C57BL6/J mice were included, and other than these were excluded from this study. Of these 22 studies, 9 studies used C57BL6/J mice; 6—Sprague Dawley rats studies; 3—CD1 mice studies; Wistar rats-2; Knockout mutant mice-1; Neonatal mouse-1 study for pharmacological evaluation. They used both genders for the study, and mostly Nitroglycerin (10 mg/kg, b.wt, i.p) chemical induction model was selected and utilized to create migraine-like conditions.

### 3.3 Intervention

By decreasing microglial and NLRP3 inflammasome activity, TREM1 regulation demonstrates potential as a therapy approach for chronic migraine. Through stimulating the Nrf2 pathway, which in turn decreases oxidative stress and neuroinflammation, NBP lessens the severity of migraines. One possible mechanism by which Wuzhuyu Decoction reduces inflammation and oxidative stress in migraine sufferers is via activating the NRF2 pathway. A possible treatment target in chronic migraine could be AMPK activation, which lowers neuroinflammation. UA has the ability to reduce migraine symptoms by reducing mitochondrial dysfunction in different parts of the brain. Vestibular dysfunction and increased fibrinogen levels are the results of NTG-induced migraines in rats. Possible migraine treatment targets include the trigeminal nucleus caudalis (TNC), which shows increased expression of PACAP and PAC1R. One novel approach to studying migraine in rats is the use of postauricular injection. In mice that have peripheral hypersensitivity caused by NTG, acupuncture at the GV16 acupoint alleviates mechanical and cold allodynia. RUT alleviates migraine symptoms by triggering the antioxidant mechanism of Nrf2. Possible relief from migraine symptoms may be achieved through the inhibition of the NGF/TRPV1/COX-2 pathway by Shaoyao Gancao Decoction. A possible therapeutic target could be the suppression of PACAP/PAC1R, since repeated intranasal PACAP treatments in CM rats cause hyperalgesia. The fact that perampanel blocks PACAP expression suggests it may be useful in treating migraines. It has been suggested that PAR2 could be a therapeutic target for migraines, as its activation and IL-6 signaling could prime NO donors. Metabolite and target influences on MXFD’s efficacy in migraine treatment provide light on areas for future investigation. Novel migraine therapy techniques may be available via BAY-117082’s modulation of the Erk/CREB/Akt pathways. One mechanism by which W146 reduces chronic NTG-induced hypersensitivity is via inhibiting S1PR1 activity. An increase in nitric oxide levels may control the pathogenesis of migraines through a variety of molecular pathways. BoNT/A may have a therapeutic function in migraines by reducing the expression of NLRP3 and IL-1β. By influencing neurotransmitter synthesis and the expression of CB1R and COX-2, Hejie Zhitong, when prescribed, may reduce migraine symptoms. A potential new approach to treating migraines could be to inhibit NLRP3, which would lessen hyperalgesia and central sensitization. It has been suggested that proinflammatory mediators such as COX-2, TNF-α, and MMP9 could be viable pharmaceutical targets in the development of migraines caused by NO.

### 3.4 Comparison

The comparison of findings across the 22 studies underscores the complexity of migraine pathophysiology and highlights the diverse approaches to intervention and treatment. Many studies focus on specific molecular pathways implicated in migraine pathophysiology, such as neuroinflammation (57, 60, 65), oxidative stress (58, 59, 61), and mitochondrial dysfunction (61, 65). These studies suggest that targeting these pathways could offer therapeutic benefits for migraine patients. Various pharmacological agents are explored across the studies, including natural compounds (58, 65), traditional Chinese medicines (59, 66), and novel drugs (62, 67). These interventions aim to modulate molecular targets associated with migraine pathogenesis, highlighting the potential of diverse treatment modalities. Studies utilize different animal models of migraine induction, including NTG-induced models (57, 62, 68, 71), GTN-induced models (31, 73), and others. Variations in animal species, strains, and induction methods contribute to the diversity of findings and insights into migraine mechanisms. Behavioral tests are commonly employed to evaluate migraine symptoms and treatment outcomes across studies, including assessments of mechanical hypersensitivity (59, 61), light aversion (61), and vestibular function (62). These assessments provide valuable insights into the efficacy of interventions in alleviating migraine-associated symptoms. Studies investigate a range of molecular targets implicated in migraine pathogenesis, such as CGRP (58, 64), NLRP3 inflammasome (57, 71), and PACAP/PAC1R signaling (62). Targeting these specific molecules offers potential avenues for developing novel migraine therapies. While the specific mechanisms vary, many interventions exert their therapeutic effects by modulating key signaling pathways associated with migraine, including the NF-κB pathway (57), Nrf2 antioxidant pathway (58, 65), and ERK/CREB/BDNF pathway (62). Understanding these mechanisms provides insights into the underlying biology of migraine and informs the development of targeted therapies. Migraine is one of several neurological illnesses studied using C57BL/6 mice in preclinical settings. Researchers frequently opt to use pre-existing models because they allow them to make use of prior knowledge and procedures, which in turn makes it easier to analyze data and compare it to other studies. Although there is not a foolproof mouse model of migraine because the condition is complex, some mouse strains, such C57BL/6, can experience migraine-like symptoms in response to certain environmental factors. Behavioral abnormalities, nociceptive reactions, and neurochemical changes similar to migraine patients may be among these symptoms. There is a gender difference in migraine prevalence, with women experiencing more attacks than men. To get a full view of the pathogenesis and treatment responses to migraines, it is essential to research both sexes. Researchers may be able to simplify trial design by minimizing any confounding effects due to hormonal fluctuations in females or study sex-specific differences in antimigraine activity by focusing on male mice in this circumstance. Migraine sensitivity and intensity are both affected by hormonal changes, especially changes in estrogen levels. Because male mice do not experience these hormonal changes, researchers can use them to their advantage when designing experiments to determine the effectiveness of antimigraine drugs.

### 3.5 Outcome

Across the research included in this review, a variety of outcome measures were observed, such as behavioral tests (*n* = 9; 40.09%) (30, 70, 74–76), Sensory sensitivity testing (28), von Frey Testing (27, 77), Light/dark test (27), Von frey test (27). To check the specific biomarker estimation ELISA (*n* = 8; 36.36%) (30, 31, 72, 74, 75, 77–79), Western blot analysis (*n* = 9; 40.09%) (28, 70, 73–75, 79–83), immunohistochemistry (*n* = 4; 18.18%) (72, 73, 75, 77, 78, 81, 82), Histopathology (27), Immunofuorescence staining (*n* = 9; 40.09%) (27, 28, 30, 31, 70, 76, 78), and for gene expression used qRT PCR (*n* = 7; 31.81%) (27, 28, 31, 70, 74, 77, 78, 80, 81, 83). The majority of researchers, around 60–70%, neglect to validate their experiments using behavioral tests, Western blotting, Immunofluorescence, and Immunohistochemistry, despite these tests being crucial for experimental validation.

The systematic review reveals a significant association between NLRP3 and MMP9 expression levels and migraine progression. Elevated levels of both NLRP3 and MMP9 are consistently observed in migraine patients compared to controls, suggesting their potential involvement in the pathophysiology of the condition. Furthermore, the review highlights the inflammatory cascade initiated by NLRP3 activation and subsequent MMP9 release as a potential mechanism underlying migraine pathogenesis. These findings underscore the importance of targeting NLRP3 and MMP9 pathways for the development of novel therapeutic strategies aimed at mitigating migraine progression and improving patient outcomes.

### 3.6 Risk of bias

**Figure 2:**
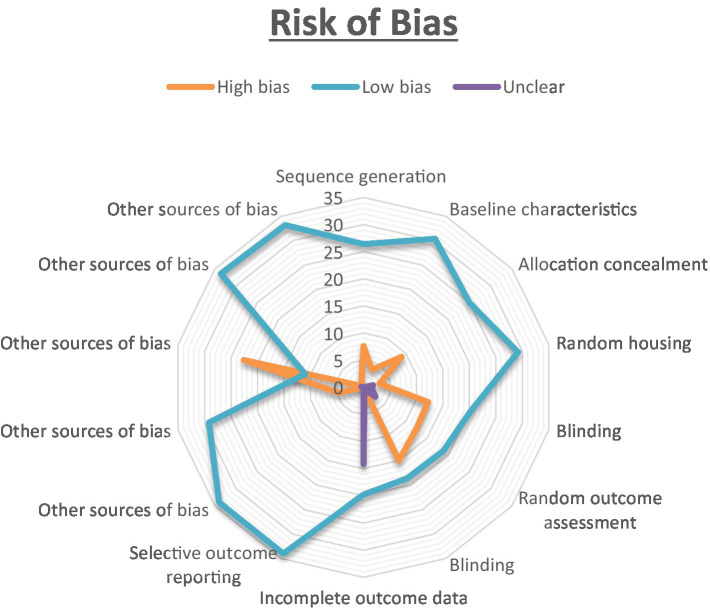
The risk of bias was analysed for all the authors. The proportion of studies with a high risk of bias categorised as ‘Yes’ is indicated in orange. The blue colour represents the proportion of studies with a low risk of bias labelled as ‘No,’ while the purple colour represents the proportion of study with an unclear response to the stated issue.

The results of the risk of bias evaluation, conducted using SYRCLE’s RoB tool, are summarized in Figure 2. Notably, there was a high risk of selection-bias, performance-bias, and detection-bias identified in the assessed studies. While all studies addressed sequence generation and allocation concealment, clarity regarding random grouping was absent in nine of them. Most studies adequately presented baseline characteristics, although three studies lacked clarity in this regard. All included studies explicitly reported random housing of animals. For the assessment of random outcome, 12 studies exhibited some degree of clarity, while it remained unclear in two studies. In most instances, there were efforts to blind investigators during behavioral outcome assessment, and histological examination was consistently blinded across all 22 studies. An overall unclear risk of bias was found in the category of attrition bias. While all studies reported complete outcome data, they did not offer clear explanations for the reasons behind or methods for handling missing data. To assess the interrater reliability among the reviewers, Cohen’s kappa statistics were employed. To ensure impartiality in the study, Mrs. Pavithra, who was blinded to it, was randomly assigned the selection of a subset of studies for evaluation. The kappa test was utilized to gauge the degree of agreement and potential discrepancies among the reviewers.

**Figure 3:**
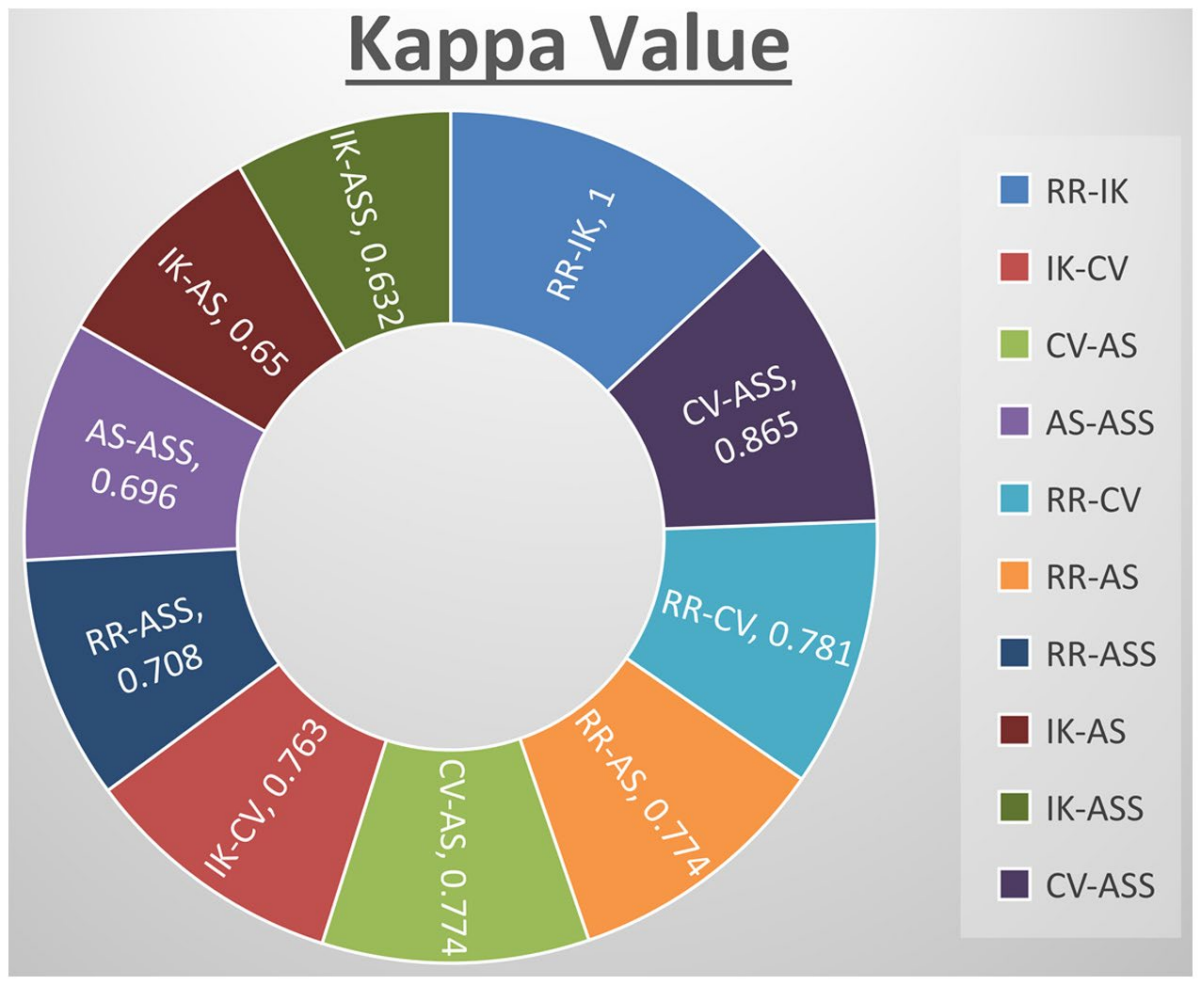
Quantify agreement by kappa value of all authors. RR-KI and VC-SA reported perfect agreement and rest others showed substantial agreement.

Kappa tests were carried out among all reviewers to validate the consistency between their assessments, revealing a substantial level of agreement. Specifically, the actual kappa score between RR and IK was calculated at 1.000 (SE of kappa = 0.000; 95% CI: 1.000–1.000; weighted kappa = 1.000), while the score between IK and CV was 0.741 (SE of kappa = 0.168; 95% CI: 0.412–1.000; weighted kappa = 0.763). CV and AS demonstrated a score of 0.745 (SE of kappa = 0.171; 95% CI: 0.570–1.000; weighted kappa = 0.836), AS and RR had a score of 0.685 (SE of kappa = 0.198; 95% CI: 0.410–1.000; weighted kappa = 0.774), AS and ASS scored 0.763 (SE of kappa = 0.150; 95% CI: 0.468–1.000; weighted kappa = 0.696), RR and CV scored 0.770 (SE of kappa = 0.217; 95% CI: 0.345–1.000; weighted kappa = 0.781), RR and AS scored 0.767 (SE of kappa = 0.214; 95% CI: 0.348–1.000; weighted kappa = 0.774), IK and AS showed a score of 0.641 (SE of kappa = 0.319; 95% CI: 0.015–1.000; weighted kappa = 0.650), RR and ASS showed a score of 0.841 (SE of kappa = 0.137; 95% CI: 0.572–1.000; weighted kappa = 0.708), IK and ASS showed a score of 0.803 (SE of kappa = 0.160; 95% CI: 0.490–1.000; weighted kappa = 0.632), CV and ASS showed a score of 0.853 (SE of kappa = 0.140; 95% CI: 0.579–1.000; weighted kappa = 0.865). Analysis of the data reveals consistent agreement among most author pairs, with some demonstrating substantial agreement, such as IK-CV (Kappa = 0.763), CV-AS (Kappa = 0.774), RR-CV (Kappa = 0.781), and RR-AS (Kappa = 0.774). However, there are instances of only fair to moderate agreement, as seen in IK-AS (Kappa = 0.65) and IK-ASS (Kappa = 0.632). Notably, the agreement between CV and ASS authors stands out with almost perfect agreement (Kappa = 0.865), contrasting the fair to moderate agreements found elsewhere. The majority of the studies included in this systematic review exhibited medium to high methodological quality, as evaluated by SYRCLE’s RoB tool. The statistical representation of the quality assessment is provided in Figure 3.

### 3.7 Quality assessment

We meticulously reviewed and assessed all reports using the standard checklist provided by SYRCLE’s-RoB tool. Cohen’s-kappa statistic was employed to ascertain the agreement between two/more raters. Among the 22 studies, it was noted that 8 of them incorporated a random component in the sequence-generation processes, whereas only 9 studies are employed C57/BL6 mice weighing between 20 and 25 g; the remaining studies used a variety of weight ranges for evaluation. The issue of investigator blinding was insufficiently addressed in all the studies, and allocation concealment was not blinded in 2 of them. Nearly all studies mentioned the random housing of animals, and 1 study provided clear documentation of blinding the outcome assessors. All the studies were ensured the random assignment of animal outcome evaluation. However, two studies inadequately addressed the handling of incomplete outcome data. There was a general lack of documentation regarding other potential causes of bias including medication dropouts, pooling, unit of analysis errors, design specific bias, and conflicts of interest.

## 4 Discussion

**Figure 4:**
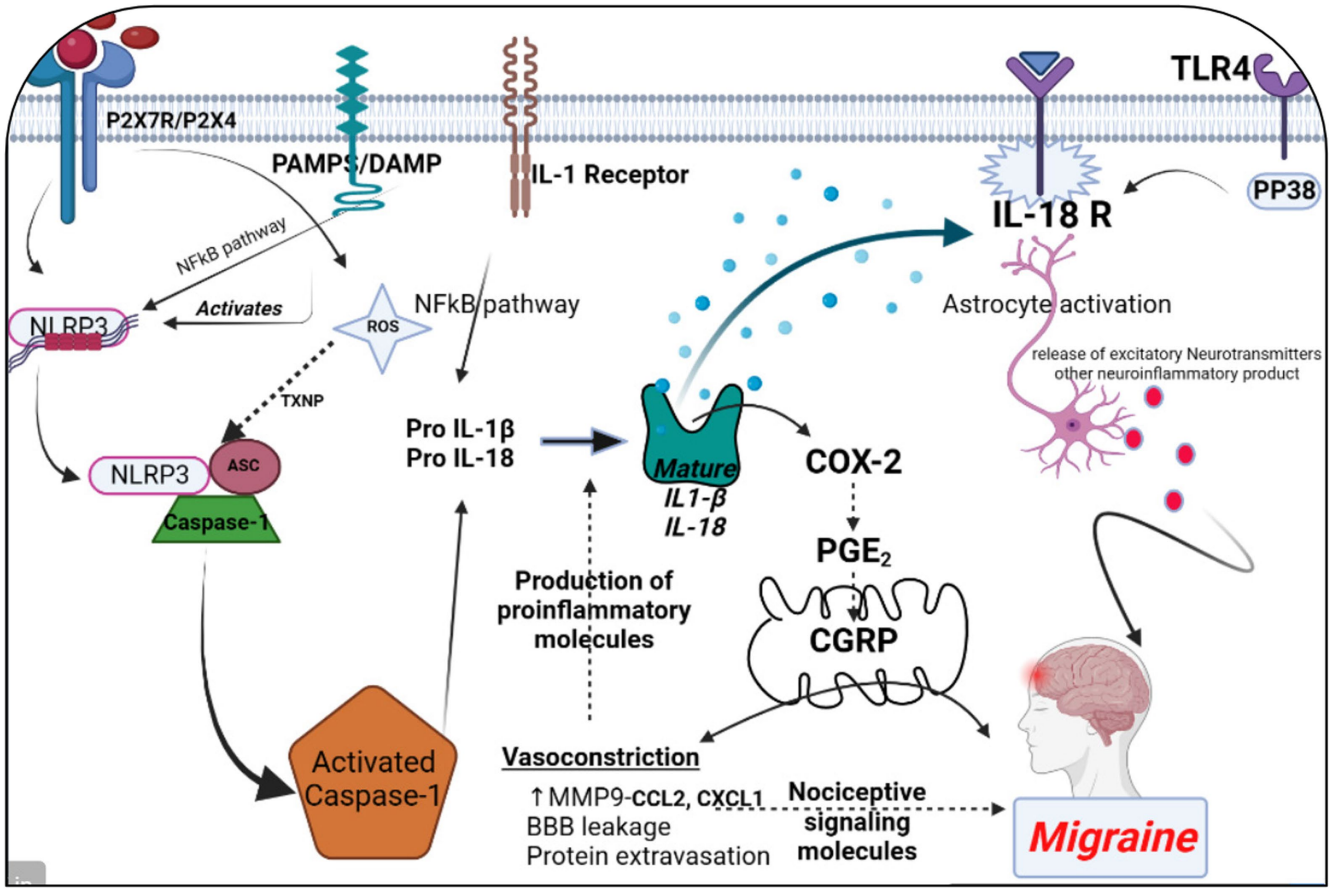
The significance of NLRP3 and MMP9 in the context of a migraine episode involves the generation and refinement of proinflammatory signaling molecules through various receptors, including P2X7R, P2X4, PAMPs or DAMP, TLR4, IL-1β, and IL-18 receptor activation. This process triggers the release of CGRP, leading to vasoconstriction by enhancing the maturation of IL-1β. Simultaneously, an elevation in MMP9 levels results in blood–brain barrier permeability and the leakage of proteins due to vasoconstriction. This phenomenon contributes to the further production of proinflammatory molecules. Additionally, astrocyte activation plays a pivotal role in the release of excitatory neurotransmitters and other neuroinflammatory substances within the brain, collectively contributing to the onset of a migraine attack. The figure was created with BioRender.com.

**Figure 5:**
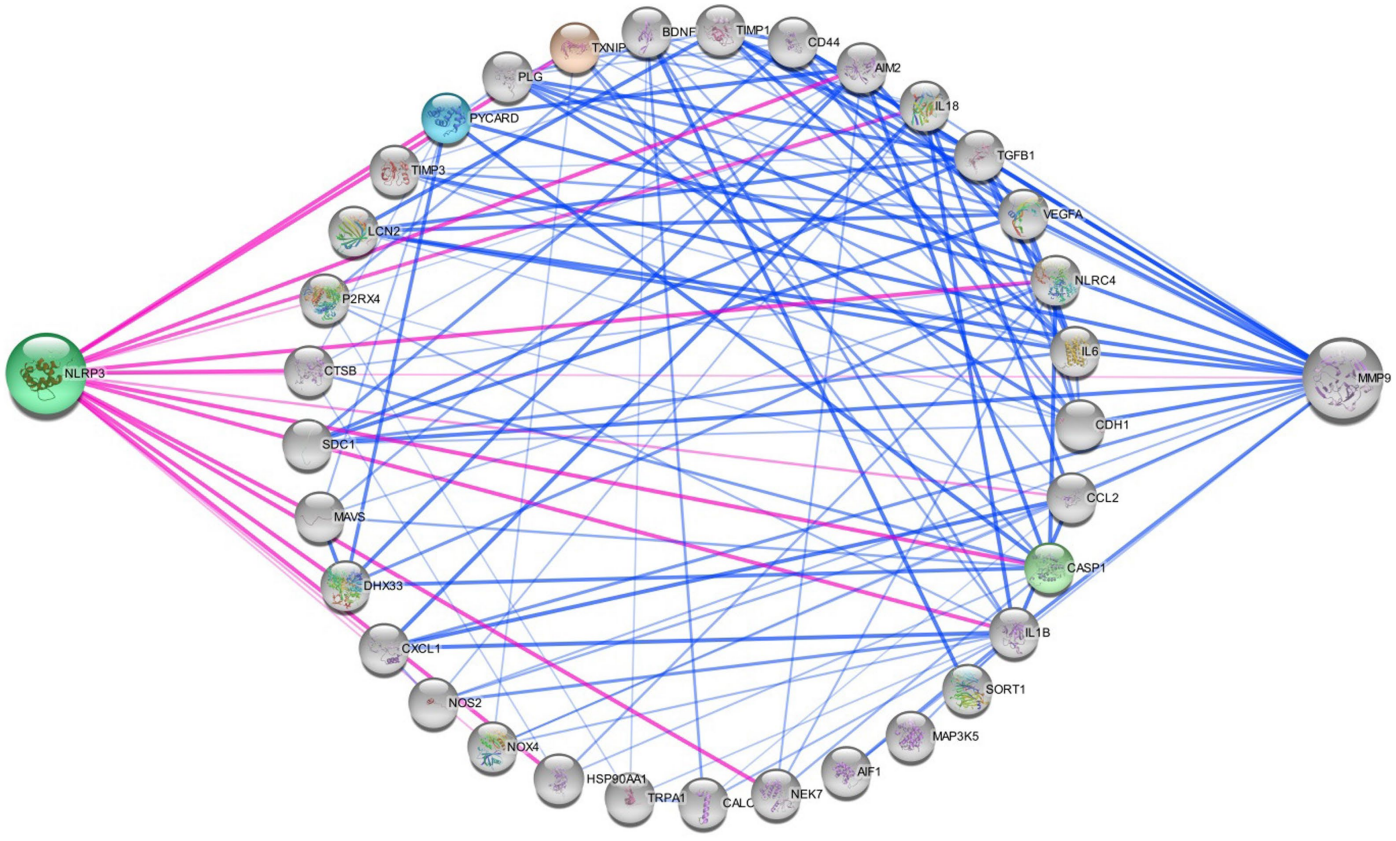
Networking of migraine targets. The figure illustrating that the different proteins interlinked with NLRP3 and MMP9 in migraine attack. There are different connecting targets of NLRP3 and MMP9 are analysed by protein–protein interaction in string database that NOX4, IL1B, P2RX4, NOS2, IL18, CCL2, TRPA1, CALCA, MAP 3 K5, TXNIP etc. are connected to migraine progression.

All 22 preclinical investigations suggested that based on the different behavioral tests, biochemical parameters, histological and immunohistochemistry reports revealed that Nitroglycerine has the capacity to damage the neuronal cells, over expression of NLRP3, Brain derived neurotrophic factor (BDNF), Nerve growth factor (NGF), RIPK1 activation, neuroinflammation, cognitive impairment, NFkB, TXNIP, ADAR3, p-ERK, c-Fos, HMGB1 and Neurotrophin-3 (NT3) may be responsible for the migraine attack. After extensive literature reviews and discussions, we concluded that the NTG-inducing model (10 mg/kg) is the most suitable for inducing the production of NLRP3, MMP9, and other proinflammatory molecules in rodents, making it widely accepted as a universal animal model for studying migraines. Elevated levels of nitric oxide, which have been associated with neuroinflammation, might regulate the movement of cellular iron. As a result, variations in iron distribution within cells could contribute to migraine pathophysiology through diverse molecular mechanisms. Brain-derived neurotrophic factor (BDNF) is linked to the modulation of pain and central sensitization. There is a growing suspicion of BDNF’s involvement in the pathophysiology of migraine and cluster headaches, primarily because of its established interaction with calcitonin gene-related peptide (84). From a clinical perspective, there is a need for increased research emphasis on NLRP3 and BDNF, as this may offer more effective solutions for migraine treatment. Studies have revealed that female mice are more susceptible to migraine compared to male mice. Through the induction of NTG (10 mg/kg) injection, researchers have observed that the end products of the NLRP3 inflammasome, specifically cytokines, elevate neurotrophic factors that play a crucial role in the pathological processes associated with the progression of migraines. Their critical findings indicate that NLRP3 is capability to mature IL1-β and is involved in the production of IL-10. Elevated levels of neurotrophic factors, particularly BDNF, may also influence NTG through the CREB/Erk/Akt pathways (27, 28, 30, 70, 75, 82). In the context of a neuropathic pain model, S1PR1 on glial cells influenced various downstream signaling pathways, including the MAPK pathways and the NLRP3 inflammasomes (85–87). S1PR1 signaling enhances the production of IL-1β by upregulating NLRP3 (70, 88–90). Wang, Yuan, and their research team provided scientific evidence indicating that the expression of the NLRP3 protein occurs in a specific manner via the P2X7R and P2X4 receptors (30, 75). These receptors can be potential targets for impeding the generation of proinflammatory molecules via NLRP3 inflammasomes in the brain, potentially aiding in the treatment of migraines (30, 75, 91). However, Fan et al. discovered that NFκB primarily plays a critical role in activating NLRP3 via TRPA1 (80). Kimono and their research team made an intriguing observation that the activation of the NLRP3 inflammasome is prompted by elevated levels of reactive oxygen species (ROS) (92). The stimulation of NLRP3 inflammasomes was linked with elevated levels of IL1β and IL18 in the brain, potentially contributing to neuroinflammation (82). An intriguing discovery is that the modulation of NLRP3-IL1β signaling plays an important role in the transition from acute to chronic neuroinflammation is represented in Figure 4, particularly driven by brain microglia. Upon activation of the Trigeminal nerve system pathway, microglia release an array of chemokines, cytokines, as well as free radicals including nitric oxide and reactive oxygen species. Nevertheless, further investigation into brain microglia is necessary to advance therapy (76). Excessively active microglia can potentially lead to neuroinflammation and tissue damage. Interestingly, both PAMP s and DAMP s are implicated in the process of neuroinflammation (93–95). PAMP s initiate the priming signaling molecule for the NLRP3inflammasome, while DAMP s serve as the second signaling molecule, fostering neuro inflammation and enabling the transmission of pain signal from primary to higher centers (79, 96). TXNIP NLRP3 plays a critical role in microglia mediated neuroinflammation. The buildup of NLRP3 leads to increased caspase1 activation and maturation (97–99). Consequently, caspase1 has the capacity to cleave pro-IL1β and pro-IL18, amplifying the proinflammatory response (78, 100–102). The Li et al. explored the presence of ADARs genes in various human tissues, which exhibit a specific down-regulation of the NLRP3 inflammasome. This discovery could potentially be identified as a novel target for migraine treatment (83, 103). MMP9 is typically regarded as an inflammatory mediator (104). Elevated MMP9 levels intensify the interconnected inflammatory processes involving CCL2 and CXCL1 (105). MMP9 is among the biomarkers that might serve as an alternative therapeutic target to disrupt BBB integrity in astrocytes, and its role in migraine pathogenesis is of significant importance to explore (31, 106–109). Several key discoveries from this systematic review were emphasized, notably the association between PX7R, RIPK, P2X4 receptor activation, NLRP3, and MMP9 in the genesis of migraines. The interconnected relationship between NLRP3 and MMP9 was depicted in Figure 5. Concentrating on these markers could offer potential solutions to alleviate the ongoing challenges faced by individuals dealing with migraines.

Activation of NLRP3 and upregulation of MMP9 contribute to neuroinflammation, which is increasingly recognized as a key factor in migraine pathogenesis. Neuroinflammation can sensitize trigeminal nociceptive pathways, leading to the generation and propagation of migraine pain. MMP9-mediated disruption of the blood–brain barrier can facilitate the entry of inflammatory cells and molecules into the brain parenchyma, exacerbating neuroinflammation and promoting migraine attacks. Both NLRP3 activation and MMP9 upregulation have been implicated in the sensitization of pain pathways, enhancing the perception of pain associated with migraine attacks and potentially contributing to the development of chronic migraine. Cortical spreading depression (CSD), a wave of neuronal depolarization followed by suppression of neuronal activity, is believed to underlie the aura phase of migraine (110). NLRP3 inflammasome activation and MMP9-mediated neuroinflammation may modulate the susceptibility to CSD, influencing the frequency and severity of migraine attacks. The roles of NLRP3 and MMP9 in migraine pathophysiology not only sheds light on the underlying mechanisms of the disease but also identifies potential therapeutic targets. Targeting these molecules or their downstream pathways could offer new strategies for the treatment and management of migraine, particularly in patients who do not respond to currently available therapies. Treatment with TREM1 modulation in C57BL/6 J male mice and BV2 cells revealed potential therapeutic insights for chronic migraine, while interventions including Dl-3-n-butylphthalide (NBP), Wuzhuyu Decoction, AMPK activator, UA, and RUT demonstrated varied mechanisms in reducing migraine severity and neuroinflammation across different mouse models and cell lines. SYRCLE’s RoB tool was used by reviewers IK, RR, ASS, CV, and AS to thoroughly assess all the studies included in the analysis. The majority of these studies effectively reported critical aspects such as sequence generation, allocation concealment, baseline characteristics, blinding, animal housing, randomization of outcome assessment, and comprehensive outcome data, which enhanced the reliability of their findings. However, it’s worth noting that a few studies failed to provide adequate details regarding blinding and the proper execution of outcome assessment randomization (*n* = 14).

Several factors led us to conclude that nitroglycerin models alone would be sufficient for our purposes. To begin, nitroglycerin-induced migraine models have shown to be a reliable way to replicate migraine symptoms in the lab, making them a popular choice for migraine studies. An effective tool for studying the biology of migraines and assessing possible treatments, this model has undergone thorough characterization and validation in clinical and preclinical investigations. The second benefit of nitroglycerin-induced migraine models is that they can reproduce both the acute and chronic phases of migraine, including the onset of headaches, pain sensitization, and the emergence of migraine-related symptoms like photophobia and nausea. Since nitroglycerin models capture all the hallmarks of migraine pathophysiology, they are ideal for investigating different facets of this condition and evaluating potential new treatments. We are aware that there are additional models for migraines; for example, the cortical spreading depression model and animal models that include inflammatory mediators or other migraine triggers, such as CGRP, and we have incorporated these into our exclusion criteria. In our work, we found that nitroglycerin models allowed us to examine migraine mechanisms in a clear and concentrated manner.

Taking over-the-counter pain relievers like ibuprofen or acetaminophen frequently for headache relief can lead to medication overuse headaches or rebound headaches. This phenomenon occurs when the body becomes dependent on the medication, and headaches recur as the medication wears off (111). Overuse of certain medications can disrupt the body’s natural pain-regulating systems and lead to increased sensitivity to pain, perpetuating a cycle of headaches. Studies have shown that individuals with obesity are more likely to experience migraines compared to those with a healthy weight. For instance, a person who is obese may have a higher likelihood of experiencing migraines triggered by hormonal changes associated with adipose tissue (112). The exact mechanisms linking obesity and migraines are not fully understood, but factors such as inflammation, adipose tissue-derived hormones, and metabolic changes may play a role in migraine development and progression. Individuals with obstructive sleep apnea may experience frequent interruptions in breathing during sleep, leading to oxygen desaturation and fragmented sleep patterns. These disruptions can trigger migraines or make existing migraines worse (113). Sleep disturbances like snoring and sleep apnea can alter neurotransmitter levels, increase inflammation, and affect the regulation of pain pathways, all of which may contribute to migraine susceptibility. A person who sustains a concussion in a car accident may develop post-traumatic headaches, which can evolve into chronic migraines over time (114). Traumatic brain injuries can cause structural and functional changes in the brain, including alterations in neuronal excitability, neurotransmitter levels, and pain processing pathways, all of which may contribute to the development and progression of migraines. Individuals who have experienced physical or sexual abuse may have a higher prevalence of migraines compared to those who have not experienced such trauma. The chronic stress associated with trauma can contribute to changes in brain chemistry and increase the risk of developing migraines (115). Traumatic experiences can lead to persistent alterations in stress response systems, neurotransmitter function, and emotional regulation, which may increase vulnerability to migraine attacks. Hypothyroidism, characterized by an underactive thyroid gland, can lead to hormonal imbalances that may influence migraine development. For instance, fluctuations in thyroid hormone levels can affect serotonin levels in the brain, which are implicated in migraine pathophysiology (116). Thyroid hormones play a role in regulating metabolism, neurotransmitter function, and vascular tone, all of which can impact migraine susceptibility and severity. Iron deficiency anemia, a common type of anemia, can result in reduced oxygen delivery to tissues, including the brain, which may trigger migraines or exacerbate existing headaches (117). Iron is essential for the synthesis of neurotransmitters and the maintenance of healthy blood vessels. Iron deficiency can lead to alterations in brain function and vascular reactivity, increasing the risk of migraine attacks. Individuals with anxiety or depression are more likely to experience migraines compared to those without these mental health conditions. Stress, dysregulated neurotransmitter levels, and altered pain processing pathways associated with anxiety and depression can contribute to migraine susceptibility and severity (118). Anxiety and depression are associated with dysregulation of the hypothalamic–pituitary–adrenal (HPA) axis, increased inflammation, and changes in serotonin and noradrenaline levels, all of which may influence migraine pathophysiology. Chronic tension-type headaches or frequent episodic headaches may precede the onset of chronic migraines in some individuals. Over time, these headaches may become more frequent and severe, transitioning into chronic migraines (119). Frequent headaches, regardless of their specific type, can lead to sensitization of pain pathways and alterations in pain processing mechanisms, increasing the likelihood of developing chronic migraines.

## 5 Limitations

### 5.1 Excluded papers

Several papers were omitted from consideration due to restricted access, as their complete texts were not freely available. This selective exclusion could potentially introduce publication bias and influence the outcomes of the review. It underscores the critical importance of comprehensive literature access to mitigate bias in systematic reviews.

### 5.2 Pooling of statistical data

The utilization of diverse animal models across the included studies precluded the feasibility of conducting a meta-analysis. This limitation notably impacts the robustness of quantitative synthesis. Emphasizing the value of narrative synthesis within the context of varied study designs is imperative. We will delve into the implications of the inability to amalgamate statistical data, thus ensuring a thorough understanding.

### 5.3 Sample size constraints

We acknowledge the constraint posed by the sample sizes within our included studies. A larger sample size could undoubtedly offer a more exhaustive comprehension of the subject matter. The necessity for forthcoming studies with expanded sample sizes to corroborate and generalize our findings will be duly emphasized.

### 5.4 Preclinical emphasis and translatability

Our focus primarily rests on preclinical investigations employing animal models, which may not fully encapsulate the intricate nature of migraine in humans. We recognize the inherent limitations in extrapolating findings from animal studies to human physiology. Hence, we underscore the imperative for translational research to bridge this gap and address the challenges in clinical applicability.

### 5.5 Gender bias consideration

The potential presence of gender bias in preclinical studies warrants attention and consideration, particularly concerning its ramifications on the generalizability of findings. We will thoroughly explore the issue of gender bias in preclinical research and its implications for comprehending migraine pathophysiology across diverse gender populations.

These adaptations and expansions within our discussion aim to enrich the critical appraisal of our research and augment the depth of understanding within the field of migraine progression. We greatly value your constructive feedback, which serves to refine the quality and relevance of our manuscript.

## 6 Highlights

This study delves into the potential roles of NLRP3 and MMP9 as biomarkers in the pathophysiology of migraine, shedding light on the underlying mechanisms of this complex neurological disorder. The study rigorously evaluates the methodological quality of 22 preclinical studies, enhancing the reliability of the findings and offering a robust foundation for further research in this field. It highlights the interconnected relationships between NLRP3, MMP9, and various other biomarkers, providing a comprehensive overview of their potential contributions to migraine attacks. The study provides valuable insights into the preclinical aspects of migraine, emphasizing the need for further research to bridge the gap between animal models and clinical applications. The study underscores the necessity for continued research in both preclinical and clinical settings to better understand the complex mechanisms of migraine and develop more effective treatments. The study touches on the gender bias observed in migraine susceptibility, emphasizing the importance of considering gender specific factors in future research and treatment strategies. The study transparently acknowledges its limitations, including small sample size, potential selection bias, and publication bias, ensuring a balanced interpretation of the findings. By identifying potential biomarkers and their roles in migraine, this study contributes to the ongoing efforts to improve migraine management and therapy, ultimately benefiting individuals worldwide who experience this disabling condition.

## 7 Conclusion and future perspective

In conclusion, this systematic review significantly advances our understanding of the intricate relationship between NLRP3 and MMP9 and their potential implications in the pathophysiology of migraine. Drawing insights from a comprehensive analysis of 22 preclinical studies utilizing specific animal models, this review sheds light on the interconnected pathways involving NLRP3 and MMP9, offering valuable clues to their involvement in migraine attacks. While the study’s rigorous evaluation of method quality and comprehensive analysis of biomarkers and behavioral tests enhance its credibility, several limitations warrant consideration. These include potential biases such as selection bias, small sample sizes, publication bias, and gender bias, which may affect the generalizability of the findings. Moreover, the reliance on animal models, while informative, may not fully capture the complexity of migraine in humans, limiting the direct applicability of the results to clinical practice. Despite these limitations, this review underscores the urgent need for further research, both in preclinical and clinical settings, to unravel the roles of NLRP3 and MMP9 in migraine pathogenesis comprehensively. Addressing the identified limitations and diversifying the scope of animal models and human studies are imperative steps toward bridging the translational gap between preclinical insights and clinical applications. In the pursuit of improved migraine management and therapy, ongoing investigations into specific biomarkers and their interplay hold immense promise. Future research endeavors should strive to transcend the confines of animal models, embracing a holistic approach that integrates preclinical findings with clinical observations. By doing so, we can aspire to develop more effective treatments and interventions, offering relief to the millions worldwide burdened by this debilitating neurovascular disorder. Future research should aim to bridge the gap between preclinical findings and clinical applications, ultimately benefiting the millions of individuals worldwide who suffers from this debilitating neurovascular disorder.

## Data availability statement

The raw data supporting the conclusions of this article will be made available by the authors, without undue reservation.

## Author contributions

RR: Conceptualization, Investigation, Writing – original draft, Writing – review & editing, Data curation, Methodology. AS: Investigation, Writing – review & editing, Conceptualization, Data curation, Methodology, Writing – original draft. SA: Data curation, Methodology, Writing – original draft. VC: Formal analysis, Investigation, Supervision, Validation, Visualization, Writing – review & editing. KI: Formal analysis, Investigation, Supervision, Validation, Visualization, Writing – review & editing.

## Footnotes

^1^ https://pubmed.ncbi.nlm.nih.gov/

^2^ https://www.graphpad.com/quickcalcs/kappa1/?K=3

## Glossary

NLRP3: Nod like receptor-3
MMP9: Matrix metalloproteinase 9
DALY: Disability-adjusted life years
RIPK1: Receptor interaction protein kinase
TNF: Tumor necrotic factor
BDNF: Brain derived neurotropic factor
NO: Nitric oxide
RAGE: Receptor for advanced glycation end product
COX: Cyclooxygenase
HMGB: High mobility group box
MAPK: Mitogen activated protein kinase
NFkB: Nuclear factor kappa B
TIMP: Tissue inhibitor of metalloproteinase
ERK: Extracellular signal-regulated kinase
CGRP: Calcitonin gene related peptide
CCK: Cholecystokinin
5-HT: Serotonin
Iba: Ionized calcium binding adaptor molecule
ROS: Reactive oxygen species
TXNIP: Thioredoxin-interacting protein
TRPA: Transient receptor potential ankyrin
P2X4: P2X purinoceptor 4
P2X7R: P2X purinoceptor 7 receptor
S1PR1: Sphingosine-1-phosphate receptor 1
STAT: Signal transducer and activator of transcription
CXCL1: The chemokine (C-X-C motif) ligand 1
CCL2: Chemokine (C-C motif) ligand 2
NGF: Nerve growth factor
NT3: Neurotrophin-3
NTG: Nitroglycerin
GTN: Glyceryl trinitrate
CCI: Chronic constriction injury
LPS: Lipopo lysaccharide
CSD: Cortical spreading depression

## References

[1] Seng EK Martin PR Houle TT. Lifestyle factors and migraine. Lancet Neurol. (2022) 21:911–21. doi: 10.1016/S1474-4422(22)00211-3 36115363

[2] Ala M Mohammad Jafari R Ala M Agbele AT Hejazi SM Tavangar SM . Sumatriptan alleviates radiation-induced oral mucositis in rats by inhibition of NF-kB and ERK activation, prevention of TNF-α and ROS release. Arch Oral Biol. (2020) 119:104919. doi: 10.1016/j.archoralbio.2020.104919, PMID: 32977152

[3] Gfrerer L Xu W Austen W Ashina S Melo-Carrillo A Longhi MS . Onabotulinumtoxin a alters inflammatory gene expression and immune cells in chronic headache patients. Brain. (2022) 145:2436–49. doi: 10.1093/brain/awab461, PMID: 34932787 PMC9337807

[4] Latif K Khan AU Izhar Ul Haque M Naeem K. Bergapten attenuates nitroglycerin-induced migraine headaches through inhibition of oxidative stress and inflammatory mediators. ACS Chem Neurosci. (2021) 12:3303–13. doi: 10.1021/acschemneuro.1c00146, PMID: 34455773

[5] Chen H Tang X Li J Hu B Yang W Zhan M . IL-17 crosses the blood-brain barrier to trigger neuroinflammation: a novel mechanism in nitroglycerin-induced chronic migraine. J Headache Pain. (2022) 23:1. doi: 10.1186/s10194-021-01374-9, PMID: 34979902 PMC8903553

[6] Peterlin BL Bigal ME Tepper SJ Urakaze M Sheftell FD Rapoport AM. Migraine and adiponectin: is there a connection? Cephalalgia. (2007) 27:435–46. doi: 10.1111/j.1468-2982.2007.01306.x, PMID: 17448181

[7] Sánchez-Robles EM Girón R Paniagua N Rodríguez-Rivera C Pascual D Goicoechea C. Monoclonal antibodies for chronic pain treatment: present and future. Int J Mol Sci. (2021) 22:10325. doi: 10.3390/ijms221910325, PMID: 34638667 PMC8508878

[8] Byun YJ Levy DA Nguyen SA Brennan E Rizk HG. Treatment of vestibular migraine: a systematic review and Meta-analysis. Laryngoscope. (2021) 131:186–94. doi: 10.1002/lary.28546, PMID: 32083732

[9] De Vries T Villalón CM Maassenvandenbrink A. Pharmacological treatment of migraine: CGRP and 5-HT beyond the triptans. Pharmacol Ther. (2020) 211:107528. doi: 10.1016/j.pharmthera.2020.107528, PMID: 32173558

[10] Humphrey PP Feniuk W Perren MJ Beresford IJ Skingle M Whalley ET. Serotonin and migraine. Ann N Y Acad Sci. (1990) 600:587–98. doi: 10.1111/j.1749-6632.1990.tb16912.x 2252337

[11] Paredes S Cantillo S Candido KD Knezevic NN. An Association of Serotonin with pain disorders and its modulation by estrogens. Int J Mol Sci. (2019) 20:5729. doi: 10.3390/ijms20225729, PMID: 31731606 PMC6888666

[12] Iyengar S Johnson KW Ossipov MH Aurora SK. CGRP and the trigeminal system in migraine. Headache. (2019) 59:659–81. doi: 10.1111/head.13529, PMID: 30982963 PMC6593989

[13] Krause DN Warfvinge K Haanes KA Edvinsson L. Hormonal influences in migraine – interactions of oestrogen, oxytocin and CGRP. Nat Rev Neurol. (2021) 17:621–33. doi: 10.1038/s41582-021-00544-2, PMID: 34545218

[14] Wattiez AS Sowers LP Russo AF. Calcitonin gene-related peptide (CGRP): role in migraine pathophysiology and therapeutic targeting. Expert Opin Ther Targets. (2020) 24:91–100. doi: 10.1080/14728222.2020.1724285, PMID: 32003253 PMC7050542

[15] Edvinsson L . CGRP receptor antagonists and antibodies against CGRP and its receptor in migraine treatment. Br J Clin Pharmacol. (2015) 80:193–9. doi: 10.1111/bcp.12618, PMID: 25731075 PMC4541967

[16] Overeem LH Raffaelli B Mecklenburg J Kelderman T Neeb L Reuter U. Indirect comparison of Topiramate and monoclonal antibodies against CGRP or its receptor for the prophylaxis of episodic migraine: a systematic review with Meta-analysis. CNS Drugs. (2021) 35:805–20. doi: 10.1007/s40263-021-00834-9, PMID: 34272688 PMC8354912

[17] Raffaelli B Neeb L Reuter U. Monoclonal antibodies for the prevention of migraine. Expert Opin Biol Ther. (2019) 19:1307–17. doi: 10.1080/14712598.2019.1671350 31550937

[18] Sacco S Amin FM Ashina M Bendtsen L Deligianni CI Gil-Gouveia R . European headache federation guideline on the use of monoclonal antibodies targeting the calcitonin gene related peptide pathway for migraine prevention – 2022 update. J Headache Pain. (2022) 23:67. doi: 10.1186/s10194-022-01431-x, PMID: 35690723 PMC9188162

[19] Vandervorst F Van Deun L Van Dycke A Paemeleire K Reuter U Schoenen J . CGRP monoclonal antibodies in migraine: an efficacy and tolerability comparison with standard prophylactic drugs. J Headache Pain. (2021) 22:128. doi: 10.1186/s10194-021-01335-2, PMID: 34696711 PMC8547103

[20] Ashina M Lanteri-Minet M Pozo-Rosich P Ettrup A Christoffersen CL Josiassen MK . Safety and efficacy of eptinezumab for migraine prevention in patients with two-to-four previous preventive treatment failures (DELIVER): a multi-arm, randomised, double-blind, placebo-controlled, phase 3b trial. Lancet Neurol. (2022) 21:597–607. doi: 10.1016/S1474-4422(22)00185-5, PMID: 35716692

[21] Barbanti P Goadsby PJ Lambru G Ettrup A Christoffersen CL Josiassen MK . Effects of eptinezumab on self-reported work productivity in adults with migraine and prior preventive treatment failure in the randomized, double-blind, placebo-controlled DELIVER study. J Headache Pain. (2022) 23:153. doi: 10.1186/s10194-022-01521-w, PMID: 36460983 PMC9716694

[22] Mcallister P Winner PK Ailani J Buse DC Lipton RB Chakhava G . Eptinezumab treatment initiated during a migraine attack is associated with meaningful improvement in patient-reported outcome measures: secondary results from the randomized controlled RELIEF study. J Headache Pain. (2022) 23:22. doi: 10.1186/s10194-021-01376-7, PMID: 35130832 PMC8903522

[23] Datta A Maryala S John R. A review of Eptinezumab use in migraine. Cureus. (2021) 13:e18032. doi: 10.7759/cureus.18032, PMID: 34540516 PMC8446121

[24] Reuter U . Headache research in 2022: advances and remaining challenges. Lancet Neurol. (2023) 22:14–5. doi: 10.1016/S1474-4422(22)00489-6, PMID: 36517156

[25] Silberstein S Diamond M Hindiyeh NA Biondi DM Cady R Hirman J . Eptinezumab for the prevention of chronic migraine: efficacy and safety through 24 weeks of treatment in the phase 3 PROMISE-2 (prevention of migraine via intravenous ALD403 safety and efficacy-2) study. J Headache Pain. (2020) 21:120. doi: 10.1186/s10194-020-01186-3, PMID: 33023473 PMC7539382

[26] Arab H.H. Abd El Aal H.A. Alsufyani S.E. El-Sheikh A.A.K. Arafa E.A. Ashour A.M. . (2022). Topiramate Reprofiling for the attenuation of cadmium-induced testicular impairment in rats: role of NLRP3 Inflammasome and AMPK/mTOR-linked autophagy. Pharmaceuticals (Basel) 15: 1402. doi: 10.3390/ph15111402 36422532 PMC9697422

[27] Filippone A Scuderi SA Basilotta R Lanza M Casili G Bova V . BAY-117082-driven NLRP3 inflammasome inhibition resolves nitro-glycerine (NTG) neuronal damage in in vivo model of migraine. Biomed Pharmacother. (2022) 156:113851. doi: 10.1016/j.biopha.2022.113851, PMID: 36252354

[28] He W Long T Pan Q Zhang S Zhang Y Zhang D . Microglial NLRP3 inflammasome activation mediates IL-1β release and contributes to central sensitization in a recurrent nitroglycerin-induced migraine model. J Neuroinflammation. (2019) 16:78. doi: 10.1186/s12974-019-1459-7, PMID: 30971286 PMC6456991

[29] Jing W Song S Sun H Chen Y Zhao Q Zhang Y . Mahuang-Fuzi-Xixin decoction reverses depression-like behavior in LPS-induced mice by regulating NLRP3 Inflammasome and neurogenesis. Neural Plast. (2019) 2019:1571392. doi: 10.1155/2019/1571392 31814820 PMC6877957

[30] Wang Y Shan Z Zhang L Fan S Zhou Y Hu L . P2X7R/NLRP3 signaling pathway-mediated pyroptosis and neuroinflammation contributed to cognitive impairment in a mouse model of migraine. J Headache Pain. (2022) 23:75. doi: 10.1186/s10194-022-01442-8, PMID: 35780081 PMC9250730

[31] Aral LA Ergün MA Bolay H. Cellular iron storage and trafficking are affected by GTN stimulation in primary glial and meningeal cell culture. Turk J Biol. (2021) 45:46–55. doi: 10.3906/biy-2009-1, PMID: 33597821 PMC7877714

[32] Karademir F Ozturk M Altunkaynak Y Doventas Y Mutluay B Basoglu Koseahmet F . Assessment of serum MMP-9, TIMP-1 levels and MMP-9/TIMP-1 ratio in migraine patients with and without aura. Ideggyogy Sz. (2022) 75:341–9. doi: 10.18071/isz.75.0341, PMID: 36218114

[33] Plinta A Tretjakovs P Svirskis S Logina I Gersone G Jurka A . Association of Body Mass Index, blood pressure, and Interictal serum levels of cytokines in migraine with and without Aura. J Clin Med. (2022) 11:5696. doi: 10.3390/jcm11195696 36233564 PMC9572946

[34] Mehndiratta MM Aggarwal V. Neurological disorders in India: past, present, and next steps. Lancet Glob Health. (2021) 9:e1043–4. doi: 10.1016/S2214-109X(21)00214-X 34273299

[35] Stovner LJ Hagen K Linde M Steiner TJ. The global prevalence of headache: an update, with analysis of the influences of methodological factors on prevalence estimates. J Headache Pain. (2022) 23:34. doi: 10.1186/s10194-022-01402-2 35410119 PMC9004186

[36] Steiner TJ Stovner LJ Jensen R Uluduz D Katsarava Z. Migraine remains second among the world's causes of disability, and first among young women: findings from GBD2019. J Headache Pain. (2020) 21:137. doi: 10.1186/s10194-020-01208-0 33267788 PMC7708887

[37] Gagandeep Singh MS Anil Kumar G Girish Rao N Prasad K Mathur P Pandian JD . The burden of neurological disorders across the states of India: the Global Burden of Disease Study 1990-2019. Lancet Glob Health. (2021) 9:e1129–44. doi: 10.1016/S2214-109X(21)00164-9 34273302 PMC8295043

[38] Alqarni MA Fayi KA Al-Sharif MN Siddiqui AF Alhazzani AA. Prevalence of migraine and non-migraine headache and its relation with other diseases in the adults of Aseer Region, Saudi Arabia. J Family Med Prim Care. (2020) 9:1567–72. doi: 10.4103/jfmpc.jfmpc_962_19 32509651 PMC7266241

[39] Li XY Yang CH Lv JJ Liu H Zhang LY Yin MY . Global, regional, and national epidemiology of migraine and tension-type headache in youths and young adults aged 15-39 years from 1990 to 2019: findings from the global burden of disease study 2019. J Headache Pain. (2023) 24:126. doi: 10.1186/s10194-023-01659-1 37718436 PMC10506184

[40] Amiri P Kazeminasab S Nejadghaderi SA Mohammadinasab R Pourfathi H Araj-Khodaei M . Migraine: a review on its history, global epidemiology, risk factors, and comorbidities. Front Neurol. (2021) 12:800605. doi: 10.3389/fneur.2021.800605 35281991 PMC8904749

[41] Steiner T.J. Jensen R. Katsarava Z. Linde M. Macgregor E.A. Osipova V. . (2019). Aids to management of headache disorders in primary care (2nd edition): on behalf of the European Headache Federation and Lifting The Burden: the Global Campaign against Headache. J Headache Pain 20:57. doi: 10.1186/s10194-018-0899-2 31113373 PMC6734476

[42] Steiner TJ Stovner LJ Vos T Jensen R Katsarava Z. Migraine is first cause of disability in under 50s: will health politicians now take notice? J Headache Pain. (2018) 19:17. doi: 10.1186/s10194-018-0846-2 29468450 PMC5821623

[43] Gupta J Gaurkar SS. Migraine: An Underestimated Neurological Condition Affecting Billions. Cureus. (2022) 14:e28347. doi: 10.7759/cureus.28347 36168353 PMC9506374

[44] Pescador Ruschel M.A. De Jesus O. "Migraine Headache," in StatPearls. (Treasure Island (FL) with ineligible companies. Disclosure: Orlando De Jesus declares no relevant financial relationships with ineligible companies.: StatPearls Publishing Copyright © 2024, StatPearls Publishing LLC; (2024)

[45] Feigin VL Stark BA Johnson CO Roth GA Bisignano C Abady GG . Global, regional, and national burden of stroke and its risk factors, 1990-2019: a systematic analysis for the Global Burden of Disease Study 2019. Lancet Neurol. (2021) 20:795–820. doi: 10.1016/S1474-4422(21)00252-0 34487721 PMC8443449

[46] Ashina M Hansen JM Do TP Melo-Carrillo A Burstein R Moskowitz MA. Migraine and the trigeminovascular system-40 years and counting. Lancet Neurol. (2019) 18:795–804. doi: 10.1016/S1474-4422(19)30185-1 31160203 PMC7164539

[47] Ashina M Saper J Cady R Schaeffler BA Biondi DM Hirman J . Eptinezumab in episodic migraine: A randomized, double-blind, placebo-controlled study (PROMISE-1). Cephalalgia. (2020) 40:241–54. doi: 10.1177/0333102420905132 32075406 PMC7066477

[48] Goadsby PJ . Trigeminal autonomic cephalalgias. Continuum (Minneap Minn). (2012) 18:883–95. doi: 10.1212/01.CON.0000418649.54902.0b 22868548

[49] Khan J Asoom LIA Sunni AA Rafique N Latif R Saif SA . Genetics, pathophysiology, diagnosis, treatment, management, and prevention of migraine. Biomed Pharmacother. (2021) 139:111557. doi: 10.1016/j.biopha.2021.111557 34243621

[50] NosedaR BursteinR. Migraine pathophysiology: anatomy of the trigeminovascular pathway and associated neurological symptoms, cortical spreading depression, sensitization, and modulation of pain. Pain. (2013) 154:S44–53. doi: 10.1016/j.pain.2013.07.02123891892

[51] Sutherland HG Albury CL Griffiths LR. Advances in genetics of migraine. J Headache Pain. (2019) 20:72. doi: 10.1186/s10194-019-1017-9 31226929 PMC6734342

[52] Suresh S Singh SA Vellapandian C. Bisphenol a exposure links to exacerbation of memory and cognitive impairment: a systematic review of the literature. Neurosci Biobehav Rev. (2022) 143:104939. doi: 10.1016/j.neubiorev.2022.104939, PMID: 36328120

[53] Hooijmans CR Rovers MM De Vries RB Leenaars M Ritskes-Hoitinga M Langendam MW. SYRCLE's risk of bias tool for animal studies. BMC Med Res Methodol. (2014) 14:43. doi: 10.1186/1471-2288-14-43, PMID: 24667063 PMC4230647

[54] Singh SA Suresh S Singh A Chandran L Vellapandian C. Perspectives of ozone induced neuropathology and memory decline in Alzheimer's disease: a systematic review of preclinical evidences. Environ Pollut. (2022) 313:120136. doi: 10.1016/j.envpol.2022.120136, PMID: 36089140

[55] Suresh S Begum RF Singh SA Chitra V. Anthocyanin as a therapeutic in Alzheimer's disease: a systematic review of preclinical evidences. Ageing Res Rev. (2022) 76:101595. doi: 10.1016/j.arr.2022.101595, PMID: 35217244

[56] Page MJ Mckenzie JE Bossuyt PM Boutron I Hoffmann TC Mulrow CD . The PRISMA 2020 statement: an updated guideline for reporting systematic reviews. BMJ. (2021) 372:n71. doi: 10.1136/bmj.n71 33782057 PMC8005924

[57] Sun S Fan Z Liu X Wang L Ge Z. Microglia TREM1-mediated neuroinflammation contributes to central sensitization via the NF-κB pathway in a chronic migraine model. J Headache Pain. (2024) 25:3. doi: 10.1186/s10194-023-01707-w 38177990 PMC10768449

[58] Liu Y Gong Z Zhai D Yang C Lu G Wang S . Unveiling the therapeutic potential of Dl-3-n-butylphthalide in NTG-induced migraine mouse: activating the Nrf2 pathway to alleviate oxidative stress and neuroinflammation. J Headache Pain. (2024) 25:50. doi: 10.1186/s10194-024-01750-1 38565987 PMC10986135

[59] Xu M Zhang J Shi Z He Z Zhao Y Ling X . Amelioration of nitroglycerin-induced migraine in mice via Wuzhuyu decoction: Inhibition of the MZF1/PGK1 pathway and activation of NRF2 antioxidant response. J Ethnopharmacol. (2024) 326:117930. doi: 10.1016/j.jep.2024.117930 38373662

[60] Lu G Xiao S Meng F Zhang L Chang Y Zhao J . AMPK activation attenuates central sensitization in a recurrent nitroglycerin-induced chronic migraine mouse model by promoting microglial M2-type polarization. J Headache Pain. (2024) 25:29. doi: 10.1186/s10194-024-01739-w 38454376 PMC10921743

[61] Xie W Li R Tang W Ma Z Miao S Li C . Proteomics profiling reveals mitochondrial damage in the thalamus in a mouse model of chronic migraine. J Headache Pain. (2023) 24:–122. doi: 10.1186/s10194-023-01646-6 PMC10478405 37667199

[62] Zhang S Azubuine J Schmeer C. A systematic literature review on the role of glial cells in the pathomechanisms of migraine. Front Mol Neurosci. (2023) 16:1219574. doi: 10.3389/fnmol.2023.1219574 37456527 PMC10347403

[63] Qi R Zhang J Diao T Yu L. The auditory function in migraine model rats induced by postauricular nitroglycerin injection. Front Neurol. (2023) 14:1259982. 38020638 10.3389/fneur.2023.1259982 PMC10630915

[64] Kim S-J Yeo J-H Yoon S-Y Roh D-H. GV16 acupoint stimulation with bee venom reduces peripheral hypersensitivity via activation of α2 adrenoceptors in a nitroglycerin-induced migraine mouse model. Integr Med Res. (2023) 12:100999. 37953754 10.1016/j.imr.2023.100999 PMC10638029

[65] Xu R. Zhang Y.W. Gu Q. Yuan T.J. Fan B.Q. Xia J.M., etal., Brain, and Behavior (2023). Alteration of neural activity and neuroinflammatory factors in the insular cortex of mice with corneal neuropathic pain. Genes Brain Behav 22:e12842. doi: 10.1111/gbb.12842 36889983 PMC10067426

[66] Luo Y Qiu Y Zhou R Zhang Y Ji X Liu Z . Shaoyao Gancao decoction alleviates the central hyperalgesia of recurrent NTG-induced migraine in rats by regulating the NGF/TRPV1/COX-2 signal pathway. J Ethnopharmacol. (2023) 317:116781. doi: 10.1016/j.jep.2023.116781 37315643

[67] Zhai Q Wang K Zhang D Chen J Dong X Pan Y. Perampanel ameliorates nitroglycerin-induced migraine through inhibition of the cAMP/PKA/CREB signaling pathway in the trigeminal ganglion in rats. Korean J Pain. (2023) 36:335. doi: 10.3344/kjp.23039 37394274 PMC10322658

[68] Mason BN Hassler SN Defea K Boitano S Vagner J Price TJ . PAR2 activation in the dura causes acute behavioral responses and priming to glyceryl trinitrate in a mouse migraine model. J Headache Pain. (2023) 24:42. doi: 10.1186/s10194-023-01574-5 37072694 PMC10114383

[69] Ge F Zhang Y Luo Y Wang C Lu Y Zhao Y . Integrating metabolomics and network pharmacology to assess the effects of Mahuang Xixin Fuzi decoction on migraine rats induced by nitroglycerin. J Pharm Pharmacol. (2024) rgae025. 10.1093/jpp/rgae025 38517943

[70] Pan Q Wang Y Tian R Wen Q Qin G Zhang D . Sphingosine-1 phosphate receptor 1 contributes to central sensitization in recurrent nitroglycerin-induced chronic migraine model. J Headache Pain. (2022) 23:25. doi: 10.1186/s10194-022-01397-w, PMID: 35144528 PMC8903593

[71] Ting K Huh A Roldan CJ. Review of trigger point therapy for the treatment of myofascial pain syndromes. J Anesthesiol Pain Therapy. (2020) 1:22–9. doi: 10.29245/2768-5365/2020/3.1112

[72] Wang X Zhao H Liu L Niu P Zhai C Li J . Hejie Zhitong prescription promotes sleep and inhibits nociceptive transmission-associated neurotransmitter activity in a rodent migraine model. Chin Med. (2020) 15:105. doi: 10.1186/s13020-020-00386-y, PMID: 33014123 PMC7526328

[73] Kim GM Jin KS Chung CS. Differential effects of corticosteroids on the expression of cyclooxygenase-2, tumour necrosis factor-alpha and matrix metalloproteinase-9 in an animal model of migraine. Cephalalgia. (2008) 28:1179–87. doi: 10.1111/j.1468-2982.2008.01667.x, PMID: 18727644

[74] Tan S Liu H Wang Y Zhu S. The molecular mechanisms associated with the effects of Propofol in a rat model of pain due to inflammation following injection with complete Freund's adjuvant. Med Sci Monit. (2019) 25:10190–7. doi: 10.12659/MSM.918420, PMID: 31889729 PMC6953440

[75] Yuan H Lu B Ji Y Meng B Wang R Sun D . Role of P2X4/NLRP3 pathway-mediated Neuroinflammation in perioperative neurocognitive disorders. Mediat Inflamm. (2022) 2022:1–9. doi: 10.1155/2022/6355805 PMC8825560 35153623

[76] Zhu T Niu J-Q Su C-J Chen J-W Zhang Y-L Luo W-F . Botulinum neurotoxin a prevents the development of nitroglycerin-induced chronic migraine via inhibition of CGRP and NLRP3 Inflammasomes in mice. Res Square. (2020). doi: 10.21203/rs.3.rs-34811/v1

[77] Knight BE Kozlowski N Havelin J King T Crocker SJ Young EE . TIMP-1 attenuates the development of inflammatory pain through MMP-dependent and receptor-mediated cell signaling mechanisms. Front Mol Neurosci. (2019) 12:220. doi: 10.3389/fnmol.2019.00220, PMID: 31616247 PMC6764257

[78] Qiu J Xie MJ. Blocking NLRP3 inflammasome expression by RAS-like protein a mitigates neuropathic pain in chronic constriction injury rat models. Trop J Pharm Res. (2021) 20:1615–21. doi: 10.4314/tjpr.v20i8.10

[79] Shao S Xu CB Chen CJ Shi GN Guo QL Zhou Y . Divanillyl sulfone suppresses NLRP3 inflammasome activation via inducing mitophagy to ameliorate chronic neuropathic pain in mice. J Neuroinflammation. (2021) 18:142. doi: 10.1186/s12974-021-02178-z, PMID: 34162415 PMC8223331

[80] Fan Y Xue G Chen Q Lu Y Dong R Yuan H. CY-09 inhibits NLRP3 Inflammasome activation to relieve pain via TRPA1. Comput Math Methods Med. (2021) 2021:1–10. doi: 10.1155/2021/9806690 PMC8380162 34426748

[81] Gursoy-Ozdemir Y Qiu J Matsuoka N Bolay H Bermpohl D Jin H . Cortical spreading depression activates and upregulates MMP-9. J Clin Invest. (2004) 113:1447–55. doi: 10.1172/JCI200421227 15146242 PMC406541

[82] Kimono D Bose D Seth RK Mondal A Saha P Janulewicz P . Host *Akkermansia muciniphila* abundance correlates with gulf war illness symptom persistence via NLRP3-mediated Neuroinflammation and decreased brain-derived neurotrophic factor. Neurosci Insights. (2020) 15:263310552094248. doi: 10.1177/2633105520942480, PMID: 32832901 PMC7440889

[83] Li Z Zhu J Wang Y. ADAR3 alleviated inflammation and pyroptosis of neuropathic pain by targeting NLRP3 in chronic constriction injury mice. Gene. (2021) 805:145909. doi: 10.1016/j.gene.2021.145909, PMID: 34419568

[84] Fischer M Wille G Klien S Shanib H Holle D Gaul C . Brain-derived neurotrophic factor in primary headaches. J Headache Pain. (2012) 13:469–75. doi: 10.1007/s10194-012-0454-5, PMID: 22584531 PMC3464472

[85] Chen H Wang J Zhang C Ding P Tian S Chen J . Sphingosine 1-phosphate receptor, a new therapeutic direction in different diseases. Biomed Pharmacother. (2022) 153:113341. doi: 10.1016/j.biopha.2022.113341, PMID: 35785704

[86] Squillace S Spiegel S Salvemini D. Targeting the Sphingosine-1-phosphate Axis for developing non-narcotic pain therapeutics. Trends Pharmacol Sci. (2020) 41:851–67. doi: 10.1016/j.tips.2020.09.006, PMID: 33010954 PMC8491165

[87] Syed SN Weigert A Brüne B. Sphingosine kinases are involved in macrophage NLRP3 Inflammasome transcriptional induction. Int J Mol Sci. (2020) 21:4733. doi: 10.3390/ijms21134733, PMID: 32630814 PMC7370080

[88] Gao P Zhang S Zhang X Xu C Chen L Fan L . S1PR1 regulates NDV-induced IL-1β expression via NLRP3/caspase-1 inflammasome. Vet Res. (2022) 53:58. doi: 10.1186/s13567-022-01078-1, PMID: 35854395 PMC9294853

[89] Hong CH Ko MS Kim JH Cho H Lee CH Yoon JE . Sphingosine 1-phosphate receptor 4 promotes nonalcoholic steatohepatitis by activating NLRP3 Inflammasome. Cell Mol Gastroenterol Hepatol. (2022) 13:925–47. doi: 10.1016/j.jcmgh.2021.12.002 34890841 PMC8810559

[90] Hou L Yang L Chang N Zhao X Zhou X Dong C . Macrophage sphingosine 1-phosphate receptor 2 blockade attenuates liver inflammation and Fibrogenesis triggered by NLRP3 Inflammasome. Front Immunol. (2020) 11:1149. doi: 10.3389/fimmu.2020.01149, PMID: 32695095 PMC7333785

[91] Hanslik KL Ulland TK. The role of microglia and the Nlrp 3 Inflammasome in Alzheimer's disease. Front Neurol. (2020) 11:570711. doi: 10.3389/fneur.2020.570711, PMID: 33071950 PMC7530640

[92] Abais JM Xia M Zhang Y Boini KM Li PL. Redox regulation of NLRP3 inflammasomes: ROS as trigger or effector? Antioxid Redox Signal. (2015) 22:1111–29. doi: 10.1089/ars.2014.5994, PMID: 25330206 PMC4403231

[93] Gray PE . A 5th type of hypersensitivity reaction: does incidental recruitment of autoreactive effector memory T-cells in response to minute amounts of PAMPs or DAMPs, underlie inflammatory episodes in the seronegative arthropathies and acute anterior uveitis? Med Hypotheses. (2009) 73:284–91. doi: 10.1016/j.mehy.2009.03.040, PMID: 19447566

[94] Pascual M Calvo-Rodriguez M Núñez L Villalobos C Ureña J Guerri C. Toll-like receptors in neuroinflammation, neurodegeneration, and alcohol-induced brain damage. IUBMB Life. (2021) 73:900–15. doi: 10.1002/iub.2510, PMID: 34033211

[95] Varki A . Since there are PAMPs and DAMPs, there must be SAMPs? Glycan “self-associated molecular patterns” dampen innate immunity, but pathogens can mimic them. Glycobiology. (2011) 21:1121–4. doi: 10.1093/glycob/cwr087, PMID: 21932452 PMC3150115

[96] Ji RR Chamessian A Zhang YQ. Pain regulation by non-neuronal cells and inflammation. Science. (2016) 354:572–7. doi: 10.1126/science.aaf8924, PMID: 27811267 PMC5488328

[97] Franchi L Eigenbrod T Muñoz-Planillo R Nuñez G. The inflammasome: a caspase-1-activation platform that regulates immune responses and disease pathogenesis. Nat Immunol. (2009) 10:241–7. doi: 10.1038/ni.1703, PMID: 19221555 PMC2820724

[98] Ismael S Nasoohi S Yoo A Ahmed HA Ishrat T. Tissue plasminogen activator promotes TXNIP-NLRP3 Inflammasome activation after hyperglycemic stroke in mice. Mol Neurobiol. (2020) 57:2495–508. doi: 10.1007/s12035-020-01893-7, PMID: 32172516 PMC9479162

[99] Kelley N Jeltema D Duan Y He Y. The NLRP3 Inflammasome: an overview of mechanisms of activation and regulation. Int J Mol Sci. (2019) 20:3328. doi: 10.3390/ijms20133328, PMID: 31284572 PMC6651423

[100] Afonina IS Müller C Martin SJ Beyaert R. Proteolytic processing of Interleukin-1 family cytokines: variations on a common theme. Immunity. (2015) 42:991–1004. doi: 10.1016/j.immuni.2015.06.003, PMID: 26084020

[101] Kordes M Matuschewski K Hafalla JC. Caspase-1 activation of interleukin-1β (IL-1β) and IL-18 is dispensable for induction of experimental cerebral malaria. Infect Immun. (2011) 79:3633–41. doi: 10.1128/IAI.05459-11, PMID: 21708993 PMC3165484

[102] Molla MD Akalu Y Geto Z Dagnew B Ayelign B Shibabaw T. Role of Caspase-1 in the pathogenesis of inflammatory-associated chronic noncommunicable diseases. J Inflamm Res. (2020) 13:749–64. doi: 10.2147/JIR.S277457, PMID: 33116753 PMC7585796

[103] Yu Q Zhao T Liu M Cao D Li J Li Y . Targeting NLRP3 Inflammasome in translational treatment of nervous system diseases: an update. Front Pharmacol. (2021) 12:707696. doi: 10.3389/fphar.2021.707696, PMID: 34526897 PMC8435574

[104] Chen Q Jin M Yang F Zhu J Xiao Q Zhang L. Matrix metalloproteinases: inflammatory regulators of cell behaviors in vascular formation and remodeling. Mediat Inflamm. (2013) 2013:928315:1–14. doi: 10.1155/2013/928315 PMC3694547 23840100

[105] Korbecki J Gąssowska-Dobrowolska M Wójcik J Szatkowska I Barczak K Chlubek M . The importance of CXCL1 in physiology and noncancerous diseases of bone, bone marrow, muscle and the nervous system. Int J Mol Sci. (2022) 23:4205. doi: 10.3390/ijms23084205, PMID: 35457023 PMC9024980

[106] Lakhan SE Kirchgessner A Tepper D Leonard A. Matrix metalloproteinases and blood-brain barrier disruption in acute ischemic stroke. Front Neurol. (2013) 4:32. doi: 10.3389/fneur.2013.00032 23565108 PMC3615191

[107] Merritt CR Cisneros IE Covarrubias-Zambrano O Stutz SJ Motamedi M Bossmann SH . Liquid biopsy-based biomarkers of inflammatory nociception identified in male rats. Front Pharmacol. (2022) 13:893828. doi: 10.3389/fphar.2022.893828, PMID: 35833018 PMC9271856

[108] Rempe RG Hartz AMS Bauer B. Matrix metalloproteinases in the brain and blood-brain barrier: versatile breakers and makers. J Cereb Blood Flow Metab. (2016) 36:1481–507. doi: 10.1177/0271678X16655551, PMID: 27323783 PMC5012524

[109] Turner RJ Sharp FR. Implications of MMP9 for blood brain barrier disruption and hemorrhagic transformation following ischemic stroke. Front Cell Neurosci. (2016) 10:56. doi: 10.3389/fncel.2016.00056 26973468 PMC4777722

[110] Costa C Tozzi A Rainero I Cupini LM Calabresi P Ayata C . Cortical spreading depression as a target for anti-migraine agents. J Headache Pain. (2013) 14:62. doi: 10.1186/1129-2377-14-62, PMID: 23879550 PMC3728002

[111] Diener HC Dodick D Evers S Holle D Jensen RH Lipton RB . Pathophysiology, prevention, and treatment of medication overuse headache. Lancet Neurol. (2019) 18:891–902. doi: 10.1016/S1474-4422(19)30146-2 31174999

[112] Rivera-Mancilla E Al-Hassany L Villalón CM Maassenvandenbrink A. Metabolic aspects of migraine: association with obesity and diabetes mellitus. Front Neurol. (2021) 12:686398. doi: 10.3389/fneur.2021.686398, PMID: 34177788 PMC8219973

[113] Tiseo C Vacca A Felbush A Filimonova T Gai A Glazyrina T . Migraine and sleep disorders: a systematic review. J Headache Pain. (2020) 21:126. doi: 10.1186/s10194-020-01192-5, PMID: 33109076 PMC7590682

[114] Defrin R . Chronic post-traumatic headache: clinical findings and possible mechanisms. J Man Manip Ther. (2014) 22:36–43. doi: 10.1179/2042618613Y.0000000053, PMID: 24976746 PMC4062350

[115] Tietjen GE Peterlin BL. Childhood abuse and migraine: epidemiology, sex differences, and potential mechanisms. Headache. (2011) 51:869–79. doi: 10.1111/j.1526-4610.2011.01906.x, PMID: 21631473 PMC3972492

[116] Spanou I Bougea A Liakakis G Rizonaki K Anagnostou E Duntas L . Relationship of migraine and tension-type headache with hypothyroidism: a literature review. Headache. (2019) 59:1174–86. doi: 10.1111/head.13600, PMID: 31310335

[117] Tayyebi A Poursadeghfard M Nazeri M Pousadeghfard T. Is there any correlation between migraine attacks and Iron deficiency Anemia? A case-control study. Int J Hematol Oncol Stem Cell Res. (2019) 13:164–71. PMID: 31649807 PMC6801325

[118] Dresler T Caratozzolo S Guldolf K Huhn J-I Loiacono C Niiberg-Pikksööt T . Understanding the nature of psychiatric comorbidity in migraine: a systematic review focused on interactions and treatment implications. J Headache Pain. (2019) 20:51. doi: 10.1186/s10194-019-0988-x, PMID: 31072313 PMC6734261

[119] Chowdhury D . Tension type headache. Ann Indian Acad Neurol. (2012) 15:S83–8. doi: 10.4103/0972-2327.100023, PMID: 23024570 PMC3444224

